# Supplementary material for: Hematopoietic stem cell transplantation leads to biochemical and functional correction in two mouse models of acid ceramidase deficiency
Source: Mol Ther. 2024 Aug 5;32(10):3402–21. doi: 10.1016/j.ymthe.2024.08.004 (PMC11489543; doi:10.1016/j.ymthe.2024.08.004)
Supplement: Document S1. Figures S1–S6 [file mmc1.pdf]

YMTHE, Volume *32*

Supplemental Information

Hematopoietic stem cell transplantation leads to biochemical and functional correction in two mouse models of acid ceramidase deficiency

Jitka Rybova, Teresa Sundararajan, Ladislav Kuchar, Theresa A. Dlugi, Petr Ruzicka, William M. McKillop, and Jeffrey A. Medin

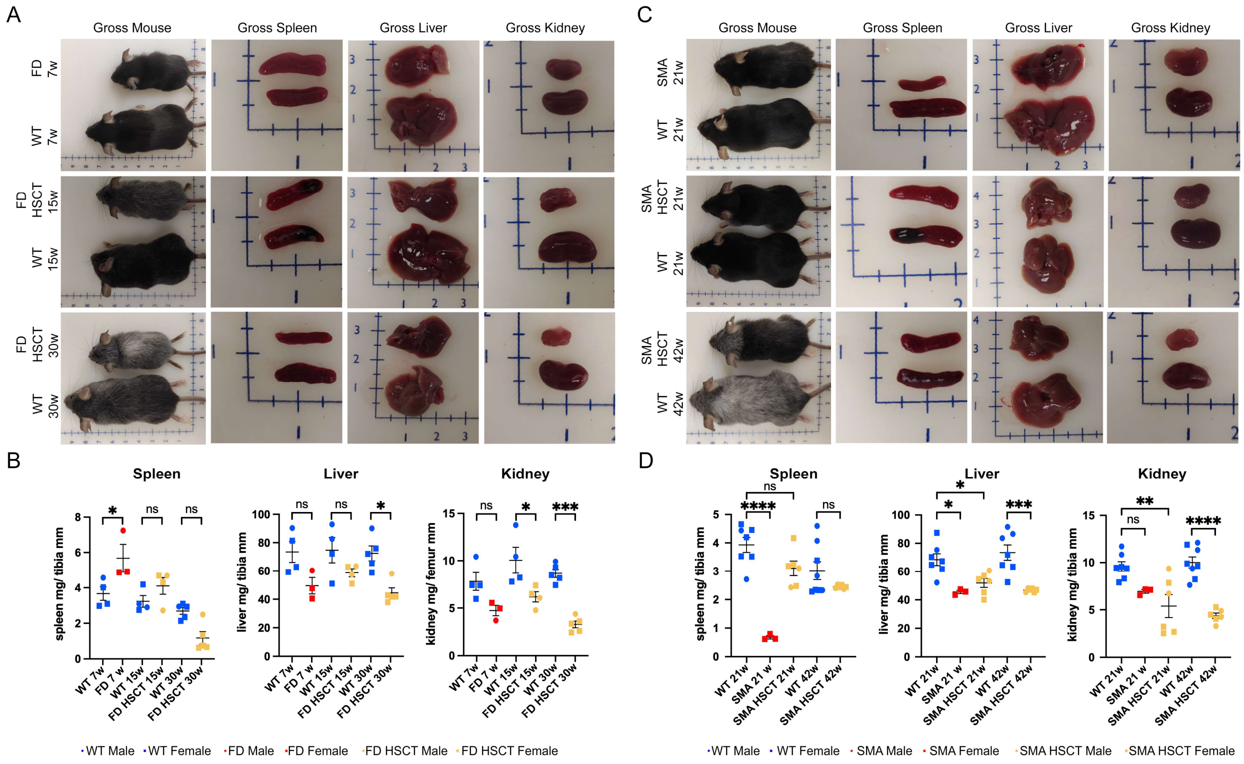

Figure S1: Body and organ weight variation following HSCT in P361R-FD mice and P361R-SMA mice

(A) Gross body, spleen, liver, and kidney images of females from untreated P361R-FD mice (7-week-old), HSC-transplanted P361R-FD mice (15- and 30-week-old), and WT mice (7-, 15- and 30-week-old).

(B) Organ weights of untreated P361R-FD mice (7-weeks-old), HSC-transplanted P361R-FD mice (15- and 30-week-old), and WT mice (7-, 15- and 30-week-old) (n = 3-5 mice per group, biological replicates). Standard error depicted. Gender combined for statistical analysis. Data analyzed using one-way ANOVA with Tukey's multiple comparison test. Significant results: *p < 0.05; ***p < 0.001, ns = not significant.

(C) Gross body, spleen, liver, and kidney images from female untreated P361R-SMA mice (21-week-old), HSC-transplanted P361R-SMA mice (21- and 42-week-old), and WT mice (21- and 42-week-old).

(D) Organ weights of untreated P361R-SMA mice (21-week-old), HSC-transplanted P361R-SMA mice (21- and 42-week-old), and WT mice (21- and 42-week-old) (n = 3-5 mice per group, biological replicates). Standard error depicted. Gender combined for statistical analysis. Data analyzed using one-way ANOVA with Tukey's multiple comparison test. Significant results: *$p < 0.05$; **$p < 0.01$; ***$p < 0.001$; ****$p < 0.0001$, ns = not significant.

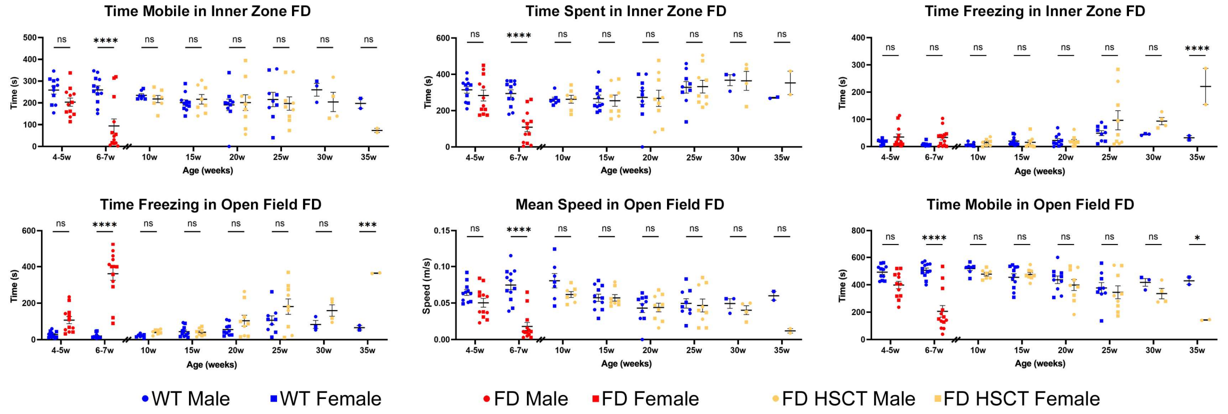

Figure S2: Open field behavioral characteristics of P361R-FD mice following HSCT

Open field characteristics including: time mobile in the inner zone, total time spent in the inner zone, time spent freezing in the inner zone, time spent freezing in the total open field, mean speed of travel in the open field, and time spent mobile in the entire open field was evaluated in untreated P361R-FD mice and WT control mice (at 4-5 and 6-7 weeks of age) in comparison to HSC-transplanted P361R-FD mice and age-matched WT littermates (n = 11-13 for each group, biological replicates). Standard error depicted. Gender combined for statistical analysis. Data analyzed using Sidak's multiple comparison test between WT and FD mice at 4-5 and 6-7 weeks of age, and between WT and HSCT treated FD mice between 10 and 35 weeks of age. Significant results: *$p < 0.05$; ***$p < 0.001$; ****$p < 0.0001$, ns = not significant.

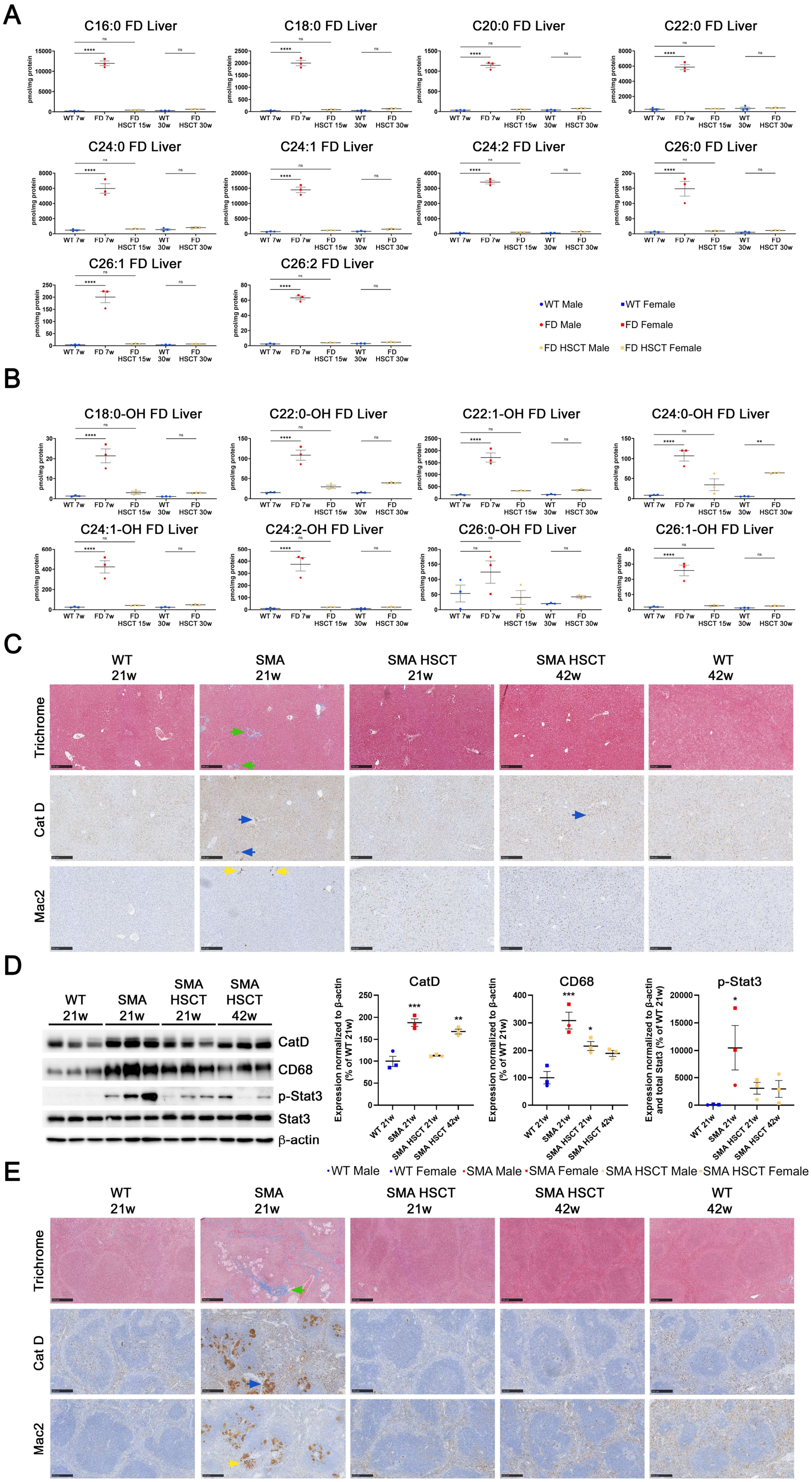

A
C16:0 FD Liver
C18:0 FD Liver
C20:0 FD Liver
C22:0 FD Liver
C24:0 FD Liver
C24:1 FD Liver
C24:2 FD Liver
C26:0 FD Liver
C26:1 FD Liver
C26:2 FD Liver
pmol/mg protein
WT 7w
FD 7w
FD HSCT 15w
WT 30w
FD HSCT 30w
WT Male
WT Female
FD Male
FD Female
FD HSCT Male
FD HSCT Female
B
C18:0-OH FD Liver
C22:0-OH FD Liver
C22:1-OH FD Liver
C24:0-OH FD Liver
C24:1-OH FD Liver
C24:2-OH FD Liver
C26:0-OH FD Liver
C26:1-OH FD Liver
C
WT 21w
SMA 21w
SMA HSCT 21w
SMA HSCT 42w
WT 42w
Trichrome
Cat D
Mac2
D
WT 21w
SMA 21w
SMA HSCT 21w
SMA HSCT 42w
CatD
CD68
p-Stat3
Stat3
β-actin
Expression normalized to β-actin (% of WT 21w)
Expression normalized to β-actin and total Stat3 (% of WT 21w)
WT 21w
SMA 21w
SMA HSCT 21w
SMA HSCT 42w
WT Male
WT Female
SMA Male
SMA Female
SMA HSCT Male
SMA HSCT Female
E
WT 21w
SMA 21w
SMA HSCT 21w
SMA HSCT 42w
WT 42w
Trichrome
Cat D
Mac2

Figure S3: Liver and spleen pathology in P361R-SMA mice following HSCT

(A-B) Quantification of selected Cer (A) and Cer-OH (B) species differing in fatty acid chain lengths in liver extracts from untreated P361R-FD mice (7-week-old), HSC-transplanted P361R-FD mice (15- and 30-week-old), and age-matched WT mice. Standard error depicted. Gender combined for statistical analysis. Data analyzed using one-way ANOVA with Tukey's multiple comparison test. Significant results: ****$p < 0.0001$, ns = not significant. ($n = 3$ mice per group, biological replicates).

(C) Liver sections from female untreated P361R-SMA mice (21-week-old), HSC-transplanted P361R-SMA mice (21- and 42-week-old), and age-matched WT mice stained by Masson Trichrome or immunostained for macrophages (Galectin-3, Mac-2) or lysosomes (Cathepsin D, CatD). Scale bars indicate 250 um.

(D) Representative immunoblots of Cathepsin D (CatD), CD68, and total and phosphorylated inflammatory protein STAT3 in liver lysates from untreated P361R-SMA mice (21-week-old), HSC-transplanted P361R-SMA mice (21- and 42-week-old), and WT mice (21-week-old) and their quantification (normalized to β-actin) relative to WT mice ($n = 3$ mice per group, biological replicates). Standard error depicted. Gender combined for statistical analysis. Data analyzed using one-way ANOVA with multiple comparison test. Significant results: *$p < 0.05$; **$p < 0.01$; ***$p < 0.001$.

(E) Spleen sections from female untreated P361R-SMA mice (21-week-old), HSC-transplanted P361R-SMA mice (21- and 42-week-old), and age-matched WT mice stained by Masson Trichrome or immunostained for macrophages (Galectin-3, Mac-2) or lysosomes (Cathepsin D, CatD) (E). Scale bars indicate 250 um.

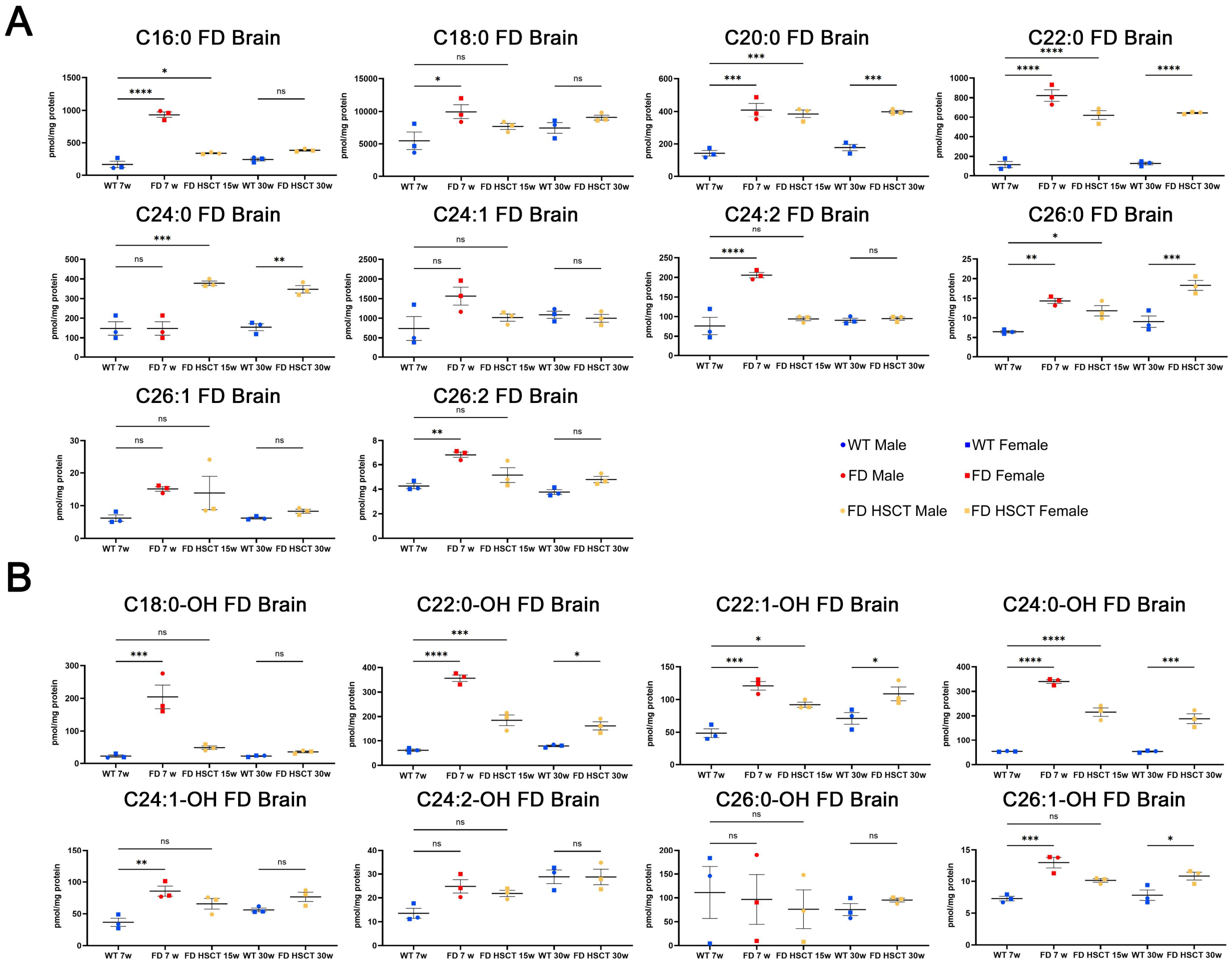

Figure S4: Improvement of brain pathology upon HSCT in both P361R-FD mice

(A, B) Quantification of Cer (A) and Cer-OH (B) species differing in fatty acid chain lengths in brain extracts from untreated P361R-FD mice (7-week-old), HSC-transplanted P361R-FD mice (15- and 30-week-old), and age-matched WT mice (n = 3 mice per group, biological replicates). Standard error depicted. Gender combined for statistical analysis. Data analyzed using one-way ANOVA with Tukey's multiple comparison test. Significant results: *$p < 0.05$; **$p < 0.01$; ***$p < 0.001$; ****$P < 0.0001$, ns = not significant.

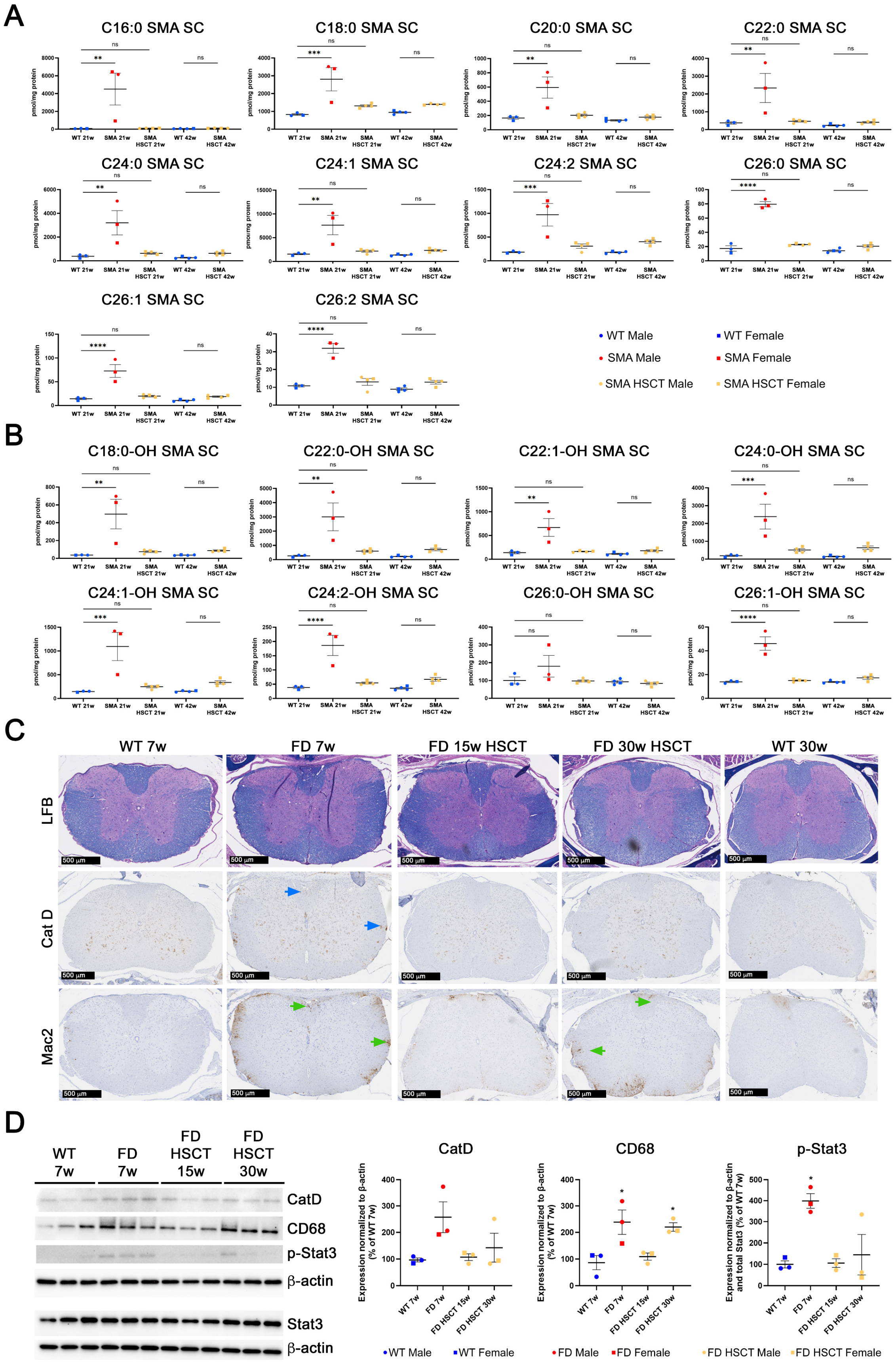

A
C16:0 SMA SC
C18:0 SMA SC
C20:0 SMA SC
C22:0 SMA SC
C24:0 SMA SC
C24:1 SMA SC
C24:2 SMA SC
C26:0 SMA SC
C26:1 SMA SC
C26:2 SMA SC
pmol/mg protein
WT 21w
SMA 21w
SMA HSCT 21w
WT 42w
SMA HSCT 42w
WT Male
WT Female
SMA Male
SMA Female
SMA HSCT Male
SMA HSCT Female
B
C18:0-OH SMA SC
C22:0-OH SMA SC
C22:1-OH SMA SC
C24:0-OH SMA SC
C24:1-OH SMA SC
C24:2-OH SMA SC
C26:0-OH SMA SC
C26:1-OH SMA SC
C
WT 7w
FD 7w
FD 15w HSCT
FD 30w HSCT
WT 30w
LFB
Cat D
Mac2
500 μm
D
WT 7w
FD 7w
FD HSCT 15w
FD HSCT 30w
CatD
CD68
p-Stat3
β-actin
Stat3
β-actin
Expression normalized to β-actin (% of WT 7w)
Expression normalized to β-actin and total Stat3 (% of WT 7w)
WT Male
WT Female
FD Male
FD Female
FD HSCT Male
FD HSCT Female

Figure S5: Normalization of mild spinal cord pathology of P361R-FD and P361R-SMA mice upon HSCT

(A, B) Quantification of Cer (A) and Cer-OH (B) species differing in fatty acid chain lengths in spinal cord (SC) extracts from untreated P361R-SMA mice (21-week-old), HSC-transplanted P361R-SMA mice (21- and 42-week-old), and WT mice (21- and 42-week-old) ($n = 3$ mice per group, biological replicates). Standard error depicted. Gender combined for statistical analysis. Data analyzed using one-way ANOVA with Tukey's multiple comparison test. Significant results: *$p < 0.05$; **$p < 0.01$; ***$p < 0.001$; ****$P < 0.0001$, ns = not significant.

(C) Spinal cord sections from female untreated P361R-FD mice (7-week-old), HSC-transplanted P361R-FD mice (15- and 30-week-old), and WT mice (7- and 30-week-old) were stained by LFB or immunostained for macrophages (Galectin-3, Mac-2) or lysosomes (Cathepsin D, CatD). Scale bars represent 500 um.

(D) Representative immunoblots of Cathepsin D (CatD), CD68, and total and phosphorylated inflammatory protein STAT3 in liver extracts from untreated P361R-FD mice (7-week-old), HSC-transplanted P361R-FD mice (15- and 30-week-old), and WT mice (7-week-old) and their quantification relative to WT mice ($n = 3$ mice per group, biological replicates). Standard error depicted. Gender combined for statistical analysis. Data analyzed using one-way ANOVA with multiple comparison test. Significant results: *$p < 0.05$.

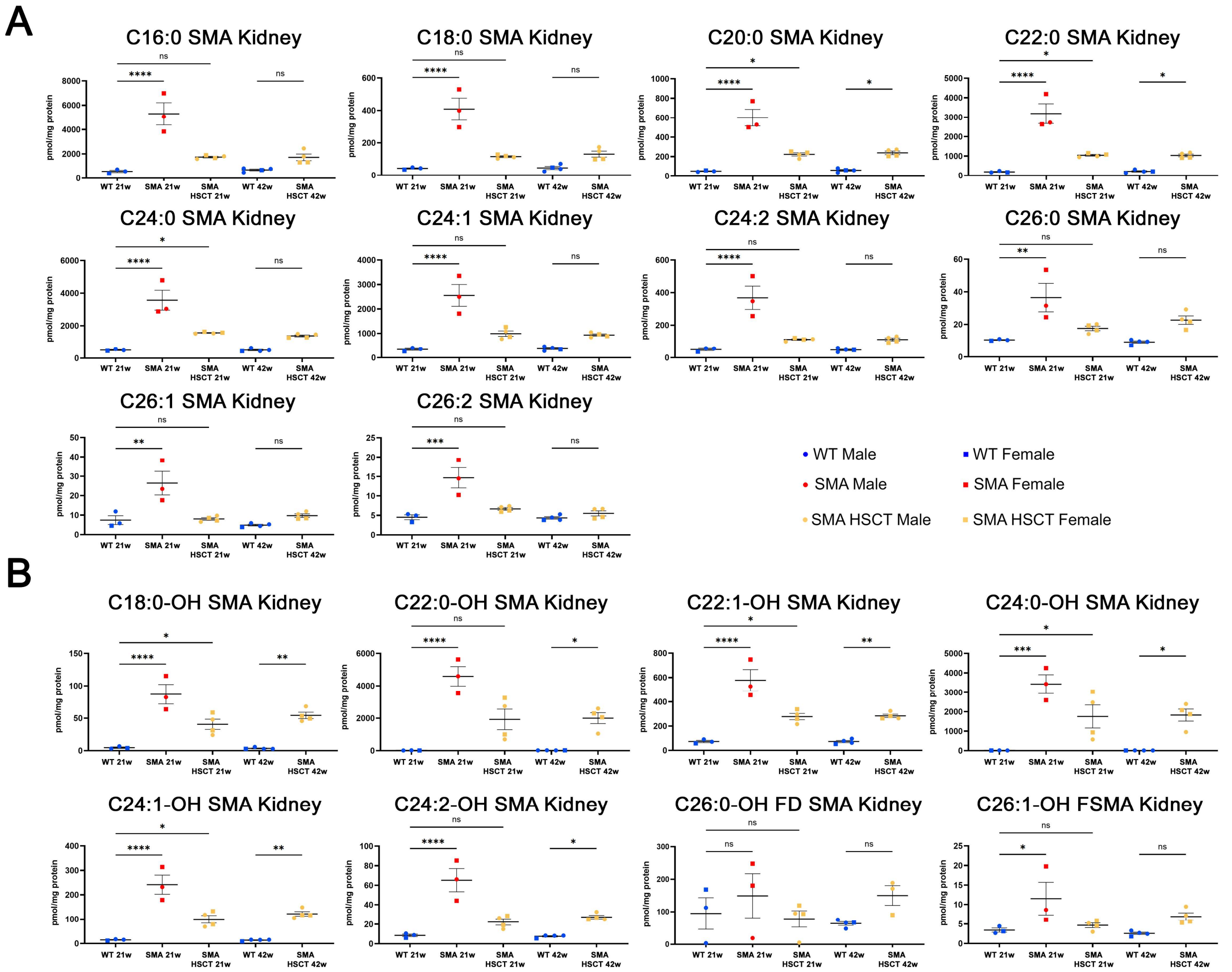

Figure S6: Quantification of ceramide species in kidney extracts from P361R-FD mice and P361R-SMA mice

(A, B) Quantification of Cer (A) and Cer-OH (B) species differing in fatty acid chain lengths in kidney extracts from untreated P361R-SMA mice (21-week-old), HSC-transplanted P361R-SMA mice (21- and 42-week-old), and WT (21- and 42-week-old) mice (n = 3 mice per group, biological replicates). Standard error depicted. Gender combined for statistical analysis. Data analyzed using one-way ANOVA with Tukey's multiple comparison test. Significant results: *p < 0.05; **p < 0.01; ***p < 0.001; ****P < 0.0001, ns = not significant.
