# Supplementary material for: Hematopoietic stem cell transplantation leads to biochemical and functional correction in two mouse models of acid ceramidase deficiency
Source: Mol Ther. 2024 Aug 5;32(10):3402–21. doi: 10.1016/j.ymthe.2024.08.004 (PMC11489543; doi:10.1016/j.ymthe.2024.08.004)
Supplement: Document S2. Article plus supplemental information [file mmc2.pdf]

Molecular Therapy
Original Article

Hematopoietic stem cell transplantation leads to biochemical and functional correction in two mouse models of acid ceramidase deficiency

Jitka Rybova,[1] Teresa Sundararajan,[1] Ladislav Kuchar,[2] Theresa A. Dlugi,[1] Petr Ruzicka,[2] William M. McKillop,[1] and Jeffrey A. Medin[1,3]

[1]Department of Pediatrics, Medical College of Wisconsin, Milwaukee, WI 53226, USA; [2]Research Unit for Rare Diseases, Department of Paediatrics and Inherited Metabolic Disorders, First Faculty of Medicine, Charles University and General University Hospital, Prague, Czech Republic; [3]Department of Biochemistry, Medical College of Wisconsin, Milwaukee, WI 53226, USA

Farber disease (FD) and spinal muscular atrophy with progressive myoclonic epilepsy (SMA-PME) are ultra-rare lysosomal storage disorders caused by deficient acid ceramidase (ACDase) activity. Although both conditions are caused by mutations in the *ASAH1* gene, clinical presentations differ considerably. FD patients usually die in childhood, while SMA-PME patients can live until adulthood. There is no treatment for FD or SMA-PME. Hematopoietic stem cell transplantation (HSCT) and gene therapy strategies for the treatment of ACDase deficiency are being investigated. We have previously generated and characterized mouse models of both FD and SMA-PME that recapitulate the symptoms described in patients. Here, we show that HSCT improves lifespan, behavior, hematopoietic system anomalies, and plasma cytokine levels and significantly reduces histiocytic infiltration and ceramide accumulation throughout the tissues investigated, including the CNS, in both models of ACDase-deficient mice. HSCT was also successful in preventing lesion development and significant demyelination of the spinal cord seen in SMA-PME mice. Importantly, we note that only early and generally pre-symptomatic treatment was effective, and kidney impairment was not improved in either model.

INTRODUCTION

Farber disease (FD) and spinal muscular atrophy with progressive myoclonic epilepsy (SMA-PME) are both autosomal-recessively inherited lysosomal storage disorders (LSDs) caused by acid ceramidase deficiency (ACDase).[1] ACDase is a lysosomal hydrolase encoded by the *ASAH1* gene that catalyzes hydrolysis of ceramide (Cer) into sphingosine and free fatty acids. ACDase deficiency leads to increased cytokine release (especially MCP-1) in plasma and Cer accumulation and histiocyte infiltration in many tissues, including the brain, liver, spleen, and thymus. Accumulation of curvilinear tubular structures known as Farber bodies has been reported in several cases.[2,3]

Currently, 107 pathogenic and 49 likely pathogenic variants have been reported for the *ASAH1* gene that are associated with distinct clinical phenotypes.[4] FD has a broad phenotypic spectrum. Children with FD and significant neurological involvement usually die early in infancy, whereas patients without or with only mild neurological findings can experience progressive joint deformation and contractures, inflammation, subcutaneous nodule development, periarticular granulomas, as well as a hoarse voice and respiratory complications. Respiratory insufficiency caused by granuloma formation in the respiratory tract and interstitial pneumonitis often leads to death in the third or fourth decade of life.[1,5]

ACDase deficiency can also result in SMA-PME, a motor neuron disease. SMA-PME represents a heterogeneous group of epilepsies, usually presenting by 2–6 years of age with difficulty walking, muscle atrophy, and tremors followed by progressive myoclonic seizures during late childhood. Mortality may result from respiratory insufficiency in early adulthood or from complications due to refractory seizures and myoclonus.[4,6,7]

There is no cure for FD or SMA-PME. Enzyme replacement therapy (ERT), substrate reduction therapy (SRT), and molecular chaperone therapy are practical choices for several LSDs including ACDase deficiencies.[1] However, these therapies are limited in their ability to relieve central nervous system (CNS) involvement due to the blood-brain barrier (BBB) preventing movement of large enzymes from the bloodstream into the brain.[8,9] Other possible treatments such as gene therapy, bone marrow transplantation (BMT), and circulating hematopoietic stem cell transplantation (HSCT) need further assessment.

The first allogenic HSCT was performed in 1957.[10] HSCT has been performed as a treatment for selected inborn errors of metabolism such as LSDs for over 30 years.[11] HSCT aims to correct the deficient

Received 9 May 2024; accepted 2 August 2024;
https://doi.org/10.1016/j.ymthe.2024.08.004.

Correspondence: Jeffrey A. Medin, Department of Pediatrics, Medical College of Wisconsin, Milwaukee, WI 53226, USA.
E-mail: jmedin@mcw.edu

 © 2024 The Author(s).
Published by Elsevier Inc. on behalf of The American Society of Gene and Cell Therapy.
This is an open access article under the CC BY license (http://creativecommons.org/licenses/by/4.0/).

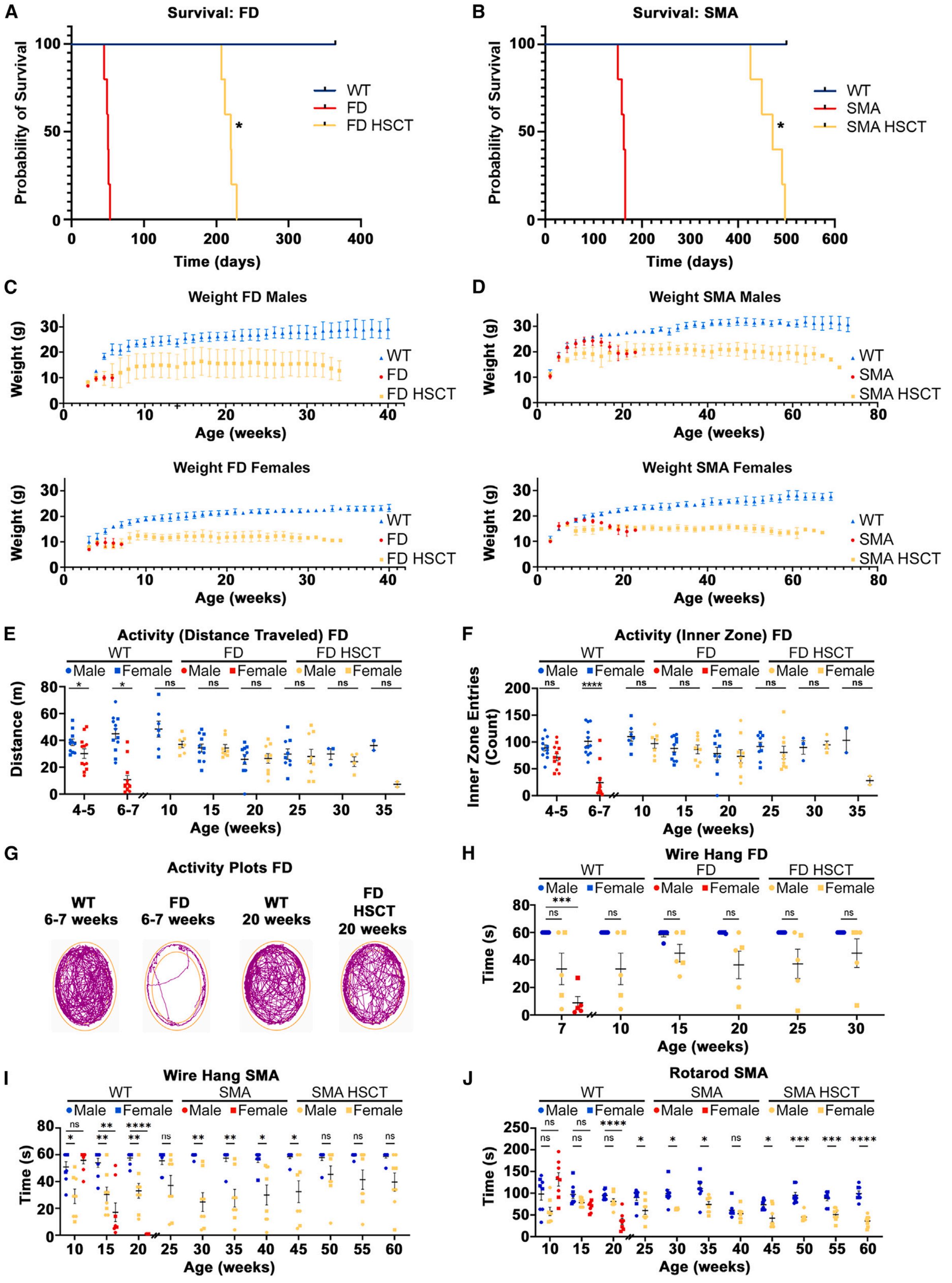

(legend on next page)

lysosomal enzyme by engrafting healthy donor hematopoietic cells.[9,11] The transplanted cells serve as a source of functional enzyme that can be taken up by enzyme-deficient host cells.[12,13] HSCT is generally a one-time procedure that results in the recipients generating their own continuous source of enzyme. HSCT is currently considered the standard of care for individuals with mucopolysaccharidosis (MPS) IH, alpha-mannosidosis, and Krabbe's disease.[13,14] HSCT is currently considered an optional treatment for Hurler/Scheie syndromes (MPS IH/S), Scheie syndrome (MPS-IS, an attenuated phenotype of MPS I), MPS II, MPS IVA, MPS VI, and MPS VII.[14] The involvement of the CNS is perilous in FD, leading to rapid deterioration and early mortality. Efforts to treat LSD patients by HSCT have resulted in favorable results in the absence of neurological manifestations,[15] possibly due to the early age of treatment.[16–18]

We have generated and thoroughly characterized mouse models of both ACDase deficiency, FD (P361R-FD)[19–25] and SMA-PME-like phenotype (P361R-SMA).[26] P361R-FD was generated by a single amino acid substitution of proline with arginine at position 361 (p.P361R) in the mouse *Asah1* gene,[25] analogous to a known ACDase deficiency causing mutation in humans (p.P362R). This mouse model recapitulates symptoms described in many FD patients, including involvement of visceral organs such as the liver,[24] severe ocular pathology,[20] chronic lung injury,[21] an abnormal skin phenotype,[23] and CNS involvement.[19] P361R-SMA was then generated by the excision of a neomycin-resistance cassette remaining in intron 12 of the P361R-FD mice. This neomycin-resistance cassette is hypothesized to partially rescue a hypomorphic allele and markedly change the natural history of these mutant mice.[26] In comparison to P361R-FD mice, P361R-SMA mice display attenuated visceral organ involvement; the dominant pathology is bladder dysfunction, a profound loss of axons and demyelination, and altered sphingolipid levels in P361R-SMA spinal cords. The fact that both of our mouse models have the same mutation but vastly different symptoms underscores the difficulty of assigning genotype-to-phenotype correlations in ACDase deficiency.[1] Lastly, although our P361R-SMA mice exhibit an increased frequency of flurothyl-induced myoclonic jerks in comparison to age-matched wild-type (WT) mice,[26] they do not appear to exhibit susceptibility to induced generalized seizures. Thus although our P361R-SMA mice display hallmarks of an SMA-like phenotype, more detailed examinations are warranted to determine the extent of possible PME-like manifestations. This is the first report of allogenic HSCT in mouse models of systemic ACDase deficiency. HSCT results in biochemical correction and functional behavioral improvements in both models of ACDase deficiency. Life span, behavioral performance on Rotarod, wire hang tests, and open-field results improved in both models of FD and SMA-PME. HSCT also prevented excessive plasma MCP-1 and interleukin (IL)-6 release, Cer accumulation, inflammation, and histiocytic infiltration in liver and spleen. HSCT also had positive effects on brain pathology in both P361R-FD and P361R-SMA mice. Moreover, HSCT prevented development of lesions, excessive demyelination, and changes in neural cell representation throughout the dorsal, lateral, and ventral columns of the spinal cord, which are typically seen in untreated P361R-SMA mice.

RESULTS

Lifespan and body-weight improvements in P361R-FD and P361R-SMA mice following HSCT

Previously generated mouse models of FD (P361R-FD)[25] and SMA-PME (P361R-SMA)[26] were used to evaluate HSCT for the treatment of these ACDase deficiencies. Hematopoietic stem cells (HSCs) isolated from bone marrow (BM) of WT mice were retro-orbitally injected into P361R-FD mice and their WT littermates (as a control) around the age of 4 weeks. We treated P361R-FD mice at 4 weeks of age because these mice usually die around the age of 7 weeks (median lifespan of 50 days; Figure 1A). We also noticed significantly higher P361R-FD mortality when injected around 5 weeks of age, likely due to disease progression. P361R-SMA mice were retro-orbitally injected between the age of 6–7 weeks, before the onset of demyelination/axon degeneration was observed.[26] P361R-SMA mice usually die several weeks later than P361R-FD mice (median lifespan of 162 days, Figure 1B). Body weights of treated and untreated P361R-FD and P361R-SMA mice and their healthy littermates were

Figure 1. Improved lifespans, body weight, and behavioral characteristics in P361R-FD mice and P361R-SMA mice following HSCT

(A and B) Kaplan-Meier survival curves for untreated and HSC-transplanted P361R-FD mice (A) and P361R-SMA mice (B) in comparison to their age-matched WT littermates (n = 5 for each genotype, biological replicates). Survival curves were compared using the Kaplan-Meier estimator (log rank test), *$p < 0.05$. (C and D) Weights of male and female untreated and HSC-transplanted P361R-FD mice (C) and P361R-SMA mice (D) over time in comparison to their age-matched WT littermates (n = 3–4 for each gender, biological replicates). Mean weights are shown for each time point with standard error indicated. (E–G) Open-field characteristics. Locomotor activity in the open field (total distance traveled) (E) and anxiety/exploration (inner zone entries) (F) evaluated in untreated P361R-FD mice and WT control mice in comparison to HSC-transplanted P361R-FD mice and age-matched WT littermates (n = 11–13 for each genotype, biological replicates). Standard error indicated. Gender combined for statistical analysis. Data analyzed using Sidak's multiple comparison test between WT and FD mice at 4–5 and 6–7 weeks of age, and between WT and HSCT-treated FD mice between 10 and 35 weeks of age. Significant results: *$p < 0.05$, **$p < 0.01$, ***$p < 0.001$, ****$p < 0.0001$. Representative activity plots for untreated P361R-FD mice (6–7 weeks of age) and HSC-transplanted P361R-FD mice (20 weeks of age) along with age-matched WT mice (G). (H and I) Neuromuscular strength evaluated by the wire hang test in untreated P361R-FD mice (6–7 weeks old) in comparison with HSC-transplanted P361R-FD mice and age-matched WT littermates (n = 5 for each genotype) (H). Neuromuscular strength evaluated by the wire hang test in untreated P361R-SMA mice in comparison with HSC-transplanted P361R-SMA mice and age-matched WT littermates (n = 8 for each genotype, biological replicates) (I). Standard error indicated. Gender combined for statistical analysis. Data analyzed using two-way repeated-measures ANOVA with Tukey's multiple comparison test. Significant results: *$p < 0.05$, **$p < 0.01$, ***$p < 0.001$. (J) Motor coordination evaluated by Rotarod in untreated P361R-SMA mice in comparison with HSC-transplanted P361R-SMA mice and age-matched WT littermates (n = 8 for each genotype, biological replicates). Standard error indicated. Gender combined for statistical analysis. Data analyzed using two-way repeated-measures ANOVA with Tukey's multiple comparison test. Significant results: *$p < 0.05$, ***$p < 0.001$, ****$p < 0.0001$.

recorded weekly (Figures 1C and 1D). Untreated P361R-FD mice (females and males) started to lose weight around 4–5 weeks of age in comparison with their WT littermates (Figure 1C). We observed a slight improvement in body-weight gain around 6–7 weeks of age in HSC-treated P361R-FD mice (Figure 1C), but these mice did not ever reach the weight of WT mice; i.e., they usually weighed 10–15 g less than control groups (Figures 1C and S1A). Median lifespan of HSCT-treated P361R-FD mice was 220 days, ~4.4 times longer than seen in untreated P361R-FD mice (Figure 1A).

We observed weight loss in P361R-SMA (females and males) upon irradiation and HSC injection at the age of 8 weeks, with more significant weight loss around 14–15 weeks of age (Figure 1D). HSC-treated P361R-SMA mice did not fully recover their weight, usually weighing 5–10 g less than their WT littermates (Figures 1D and S1C), but displayed a lengthened median lifespan of 471 days, about three times that of untreated P361R-SMA mice (median lifespan 162 days, Figure 1B).

Behavioral improvements in P361R-FD and P361R-SMA mice following HSCT

Treated and control P361R-FD and P361R-SMA mice underwent behavioral analyses to assess function following HSCT (Figures 1E–1J). While 4- to 5-week-old P361R-FD mice performed similarly to WT mice in the open field, by 7 weeks of age, untreated P361R-FD mice showed statistically significant impairment in open-field characteristics (Figures 1E, 1F, and S2A), suggesting treatment began before any obvious functional impairment had occurred. Progressively impaired locomotor activity (evaluated by distance traveled in the open field) in 7-week-old untreated P361R-FD mice (Figure 1E, red) normalized following HSCT through the age of 35 weeks (Figure 1E, orange), around the age when treated P361R-FD mice usually died. Significantly impaired anxiety/exploration (open-field inner zone entries) in 7-week-old untreated P361R-FD mice (Figure 1F, red) was also normalized following HSCT (Figure 1F, orange). Representative track plots from open-field testing conducted on 20-week-old mice showed improved anxiety and exploration in treated P361R-FD mice (Figure 1G). Untreated P361R-FD mice showed progressive impairment in other open-field characteristics, including time mobile, time spent freezing, mean traveling speed, and total time spent traveling, as well as inner-zone total time and time spent freezing (Figure S2). All these characteristics were normalized following HSCT in P361R-FD mice up until the age of 30 weeks, near the end of their lives (Figure S2).

Decreased neuromuscular strength, as evaluated by the wire hang test, was observed in 7-week-old untreated P361R-FD mice in comparison with age-matched controls (Figure 1H). HSCT led to significantly improved wire hang results with lower, but not statistically different, scores from that of WT mice (Figure 1H).

Hanging ability and Rotarod tests were used to monitor muscle strength and motor coordination, respectively, in P361R-SMA mice. Untreated P361R-SMA mice exhibited progressive defects in both characteristics over time in comparison with WT mice[26] (Figures 1I and 1J). No obvious changes among untreated P361R-SMA and WT groups were observed in younger animals before 10 weeks of age, suggesting that, similarly to the P361R-FD mice, HSCT treatment was initiated before functional impediment occurred in P361R-SMA mice. HSCT in P361R-SMA mice resulted in significantly improved neuromuscular strength over time (Figure 1I, orange) as, by 20 weeks of age, untreated P361R-SMA mice were unable to sufficiently hold the wire (Figure 1I, red). HSCT-treated P361R-SMA mice also showed improvements in motor coordination as tested by Rotarod (Figure 1J, orange) until the age of 50 weeks, when their scores were similar to those seen in untreated P361R-SMA mice at the age of 20 weeks (Figure 1J, red).

Hematopoietic system improvements in both P361R-FD and P361R-SMA mice following HSCT

Excessive plasma cytokine release is a well-known observation in both FD mice and patients.[25,27] Here, whole blood from P361R-FD, P361R-SMA, and their age-matched WT littermates was collected every 7 weeks post HSCT and tested for MCP-1 and IL-6 in plasma (Figures 2A and 2B, orange). Untreated mutant mice were included for comparison (red). Gradual increases of MCP-1 and IL-6 levels were seen in untreated mutant mice of both genotypes (Figures 2A and 2B, red) in comparison with WT mice (blue). Transplantation of HSCs resulted in complete normalization of both MCP-1 and IL-6 cytokines to control levels at the first time point measured in both P361R-FD and P361R-SMA mice. Values remained stable through the last time point evaluated (32-week-old P361R-FD mice and 55-week-old P361R-SMA mice), close to the time when treated mutant mice started to die (Figures 2A and 2B orange).

For analysis of whole-blood characteristics, histology, mass spectrometry, and western blots, we sacrificed HSCT-treated P361R-FD mice and their age-matched littermates at 15 and 30 weeks for tissue and whole-blood collections, respectively. HSCT-treated P361R-SMA mice were sacrificed at the ages of 21 and 42 weeks for the same collections. We used untreated mutants near their disease end stage for comparison (7 weeks in the case of P361R-FD mice and 21 week in the case of P361R-SMA mice).

Complete blood count (CBC) pathology was previously reported in P361R-FD mice.[1,25] In this study, we confirmed those results as we found a decreased percentage of lymphocytes, reticulocyte hemoglobin equivalent (Ret-HE), mean corpuscular hemoglobin (MCH), and reticulocyte count (Ret #), along with increased percentages of monocytes and neutrophils (Figure 2C, red) in comparison with values obtained from WT mice (blue). White blood cell (WBC) counts and hemoglobin levels (HGB) were in the normal range. Interestingly, none of the blood characteristics we studied were pathologically altered in 15-week-old P361R-FD mice following HSCT (Figure 2C, orange). Ret-HE and MCH were the only two blood characteristics evaluated that were not normalized to WT values at 30 weeks of age in P361R-FD mice after HSCT, even though they

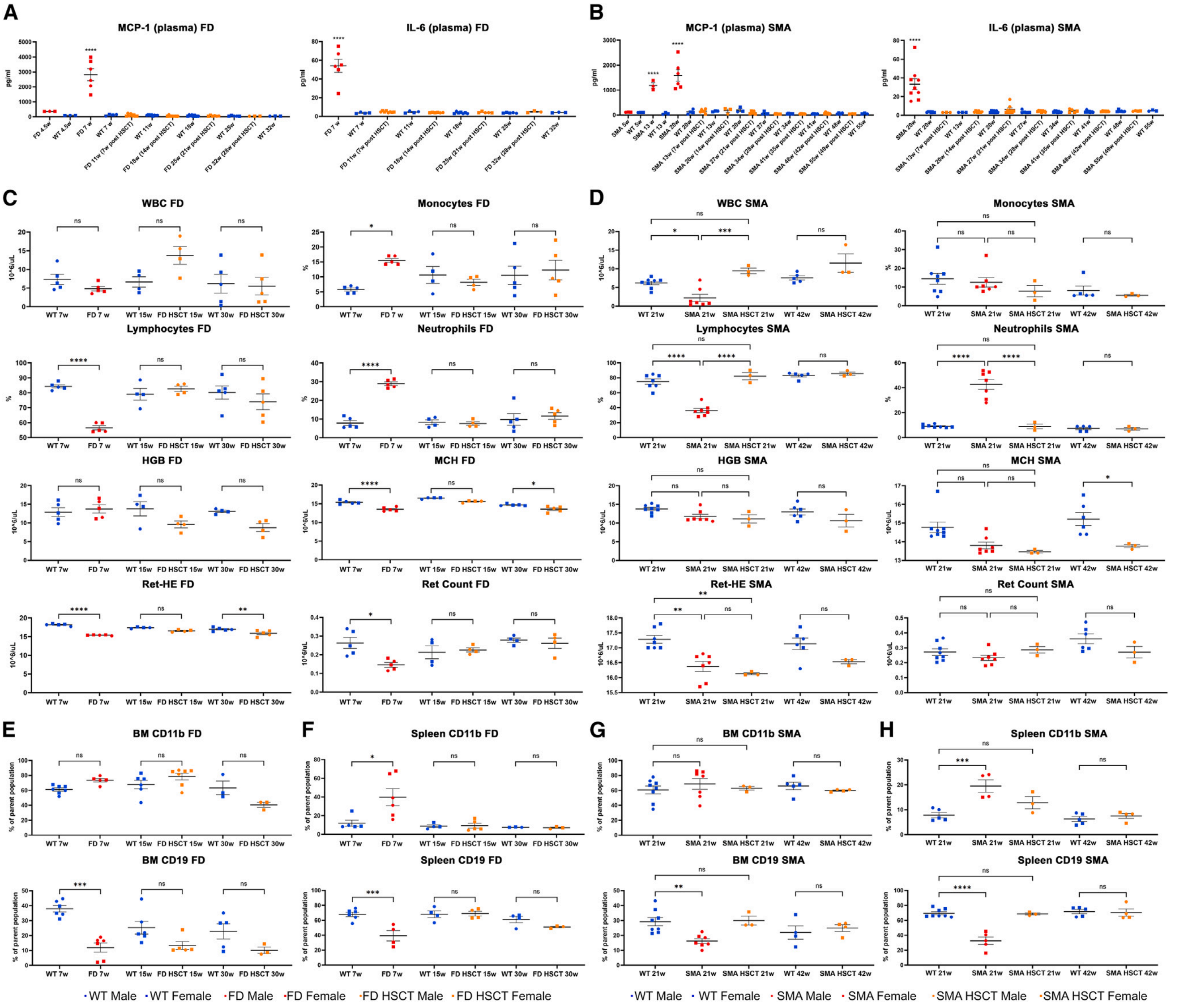

Figure 2. Normalization of plasma cytokines and selected blood characteristics in both P361R-FD mice and P361R-SMA mice following HSCT

(A) Plasma MCP-1 and IL-6 in untreated P361R-FD mice (4.5 and 7 weeks of age, red), HSC-transplanted P361R-FD mice (collected every 7 weeks post transplantation, orange), and age-matched WT control mice (blue) measured by ELISA (n = 3–9). Error bars show standard error. Gender combined for statistical analysis. Data analyzed by one-way ANOVA and Tukey's multiple comparison test. Significant results: ****$p < 0.0001$. (B) Plasma MCP-1 and IL-6 in untreated P361R-SMA mice (5, 13, and 20 weeks of age, red), HSC-transplanted P361R-SMA mice (collected every 7 weeks post transplantation, orange), and age-matched WT control mice (blue) measured by ELISA (n = 3–8). Error bars show standard error. Gender combined for statistical analysis. Data analyzed by one-way ANOVA and Tukey's multiple comparison test. Significant results: ****$p < 0.0001$. (C and D) Selected blood counts from untreated P361R-FD mice (7 weeks old), HSC-transplanted P361R-FD mice (15 and 30 weeks old), and age-matched WT mice (n = 4–5) (C). Selected blood counts from untreated P361R-SMA mice (21 weeks old), HSC-transplanted P361R-SMA mice (21 and 42 weeks old), and age-matched WT control mice (blue) (n = 3–8, biological replicates) (D). Selected blood counts included white blood cell (WBC), monocytes, lymphocytes, neutrophils, hemoglobin (HGB), mean corpuscular hemoglobin (MCH), reticulocyte hemoglobin equivalent (Ret-HE), and reticulocyte count (Ret). Gender combined for statistical analysis. Error bars show standard error. Data analyzed using two-way ANOVA and Tukey's multiple comparison test. Significant results: *$p < 0.05$; **$p < 0.01$; ****$p < 0.0001$; ns, not significant. (E and F) CD11b+ macrophages and CD19+ B cells in bone marrow (BM) (E) and spleen (F) cells in untreated P361R-FD mice (7 weeks old), HSC-transplanted P361R-FD mice (15 and 30 weeks old), and age-matched WT control mice (n = 3–7, biological replicates). Error bars show standard error. Gender combined for statistical analysis. Data analyzed using two-way ANOVA and Tukey's multiple comparison test. Significant results: **$p < 0.01$; ***$p < 0.001$; ns, not significant. (G and H) CD11b+ macrophages and CD19+ B cells in BM (G) and spleen cells (H) in untreated P361R-SMA (21 weeks old), HSC-transplanted P361R-SMA mice (21 and 42 weeks old), and age-matched WT control mice (n = 3–8, biological replicates). Error bars show standard error. Gender combined for statistical analysis. Data analyzed using two-way ANOVA and Tukey's multiple comparison test. Significant results: **$p < 0.01$; ***$p < 0.001$; ****$p < 0.0001$; ns, not significant.

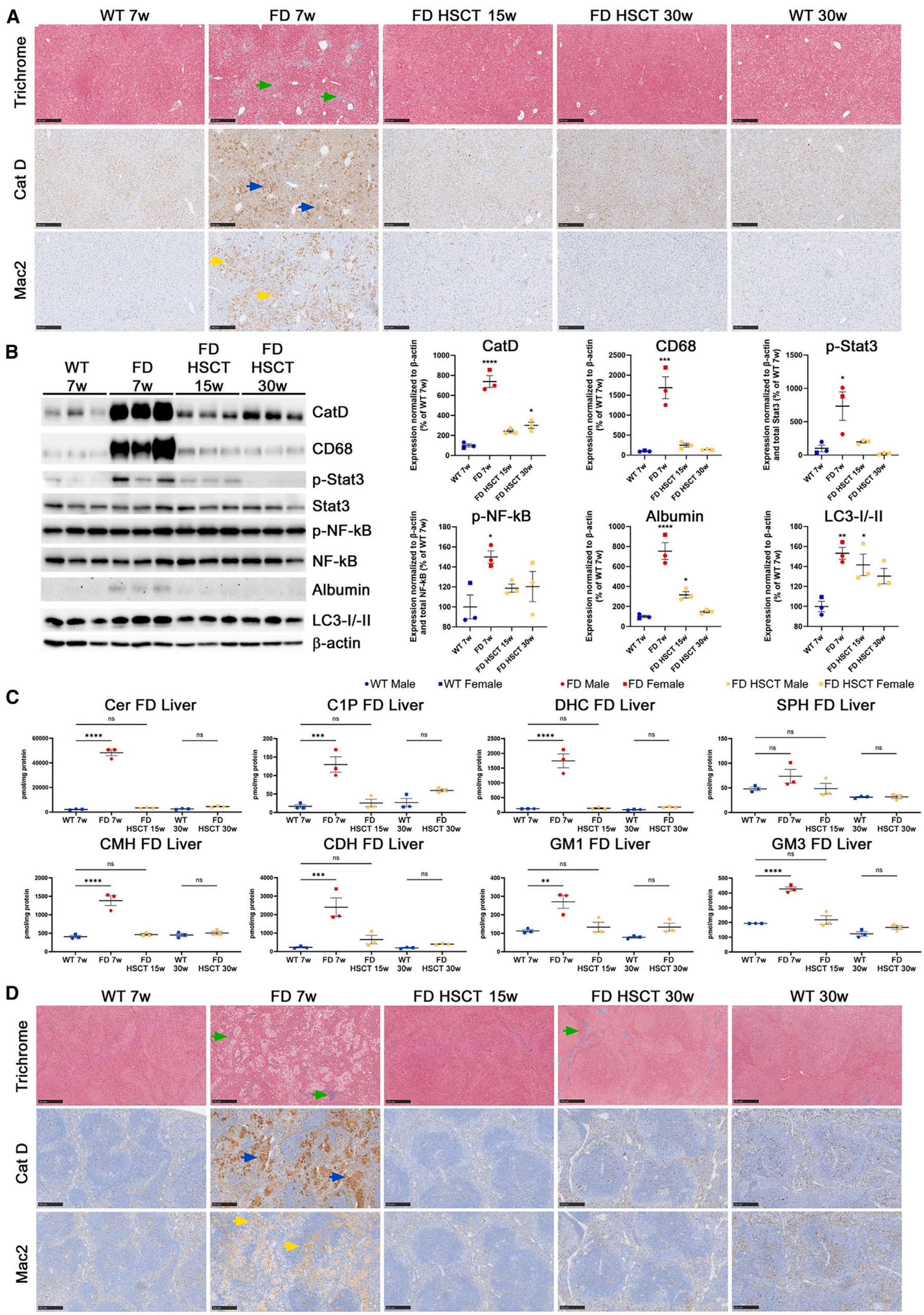

(legend on next page)

were significantly improved in comparison with untreated mutant mice at end-stage and age-matched WT mice.

CBC analysis has not previously been studied in P361R-SMA mice. Similar to P361R-FD mice, untreated P361R-SMA mice showed a decreased percentage of lymphocytes and Ret-HE and an increased percentage of neutrophils compared to WT littermates (Figure 2D, red). Untreated P361R-SMA mice showed a significantly decreased WBC count. Other blood characteristics were in the normal range. These results, along with a complete normalization of all studied blood characteristics to control values after HSCT (Figure 2D, orange), suggest a milder disturbance of the hematopoietic system in P361R-SMA mice than in P361R-FD mice.

A significant reduction of immature B and T cells and their progenitors in the BM and thymus isolated from the *Asah1*$^{P361R/P361R}$ mouse model of FD has previously been reported,[22] but this has not been studied in P361R-SMA mice. Here, we investigated CD11b+ monocyte/macrophages and CD19+ B cells in the BM and spleen by flow cytometry in both untreated and treated P361R-FD and P361R-SMA mice (Figures 2E–2H). CD11b+ monocytes/macrophages were slightly increased in the BM, but not significantly, in both P361R-FD and P361R-SMA mice and were unaltered following HSCT (Figures 2E and 2G). The same untreated mutant mice displayed significantly increased CD11b+ monocyte/macrophages in spleens from both genotypes, and this was normalized following HSCT (Figures 2F and 2H). Both untreated P361R-FD and P361R-SMA mice displayed reduced CD19+ B cells in BM and spleen, and this was also normalized following HSCT at all time points studied (Figures 2E–2H).

HSCT results in normalization of spleen and liver size, inflammation markers, lysosomal accumulation, and macrophage infiltration

Prominent deterioration of several organs, such as liver, spleen, skin, brain, and lung, have been reported in the *Asah1*$^{P361R/P361R}$ mouse model of FD.[19,21,23–25] P361R-SMA mice predominantly demonstrated spinal cord involvement.[26] Here, we focused on an analysis of the liver, spleen, kidney, brain, and spinal cord to determine whether HSCT was beneficial for these organs.

Histological analyses of the liver (Figure 3A) demonstrated significant tissue disruption, fibrosis (Masson trichrome, green arrows), massive accumulation of lysosomal clusters (Cathepsin D [CatD], blue arrows), and macrophage infiltration (Mac2, yellow arrows) in liver tissue from 7-week-old untreated P361R-FD mice compared to WT.[24,26] Despite a slight reduction in liver weights at the age of 30 weeks when compared to length of the tibia (Figures S1A and S1B), HSCT resulted in prevention of histological pathology observed in untreated mice (Figure S3A). Minimal fibrosis and reduced Mac2 histiocyte infiltration within liver parenchyma was observed (Figure S3A) in HSCT-treated P361R-FD mice. Thirty-week-old HSCT-treated P361R-FD mice were also free of histological pathology compared to WT. CatD staining was slightly increased in 30-week-old treated P361R-FD mice, but not to the extent observed in untreated animals (Figure S3A). Western blot analyses of liver lysates from untreated and treated P361R-FD mice confirmed prevention of lysosomal marker CatD and macrophage marker CD68 accumulation in P361R-FD mice following HSCT (Figure 3B). HSCT treatment led to a significant decrease in phosphorylated inflammatory proteins STAT3 and nuclear factor κB (NF-κB) in the same liver lysates in comparison with untreated P361R-FD mice (Figure 3B). These phosphorylated proteins act as part of the inflammatory IL-6/JAK/signal transducer and activator of transcription 3 (STAT3) and noncanonical NF-κB signaling pathways and have previously been found to be increased in *Asah1*$^{P361R/P361R}$ mice.[23,24] Autophagy markers LC3-I/-II and albumin were normalized in liver lysates from P361R-FD mice following HSCT (Figure 3B). Reduction of these proteins was most prominent in older 30-week-old mice in comparison with 15-week-old treated mutants (Figure 3B).

Accumulation of Cer, as well as the buildup of other sphingolipids involved in Cer metabolic pathways, has been widely reported in various organs from ACDase-deficient mice.[20,21,23,24,26,28] Mass spectrometry quantification of sphingolipids in liver extracts from untreated and treated P361R-FD mice and their age-matched WT controls confirmed massive accumulation of total Cer along with statistically significant accumulation of ceramide-1-phosphate (C1P), dihydroceramide (DHC), monohexosylceramide (CMH), dihexosylceramide (CDH), along with GM1 and GM3 gangliosides in untreated P361R-FD mice compared to age-matched WT mice.

Figure 3. Improved liver and spleen pathology in P361R-FD mice following HSCT

(A) Liver sections from females of untreated P361R-FD mice (7 weeks old), HSC-transplanted P361R-FD mice (15 and 30 weeks old), and age-matched WT mice were stained using Masson trichrome or immunostained for macrophages (Mac2, Galectin-3) or lysosomes (Cathepsin D [CatD]) (A). Scale bars, 250 μm. (B) Representative immunoblots of CatD, CD68, albumin, LC3-I/-II, and total and phosphorylated inflammatory proteins STAT3 and NF-κB in liver lysates from untreated P361R-FD mice (7 weeks old), HSC-transplanted P361R-FD mice (15 and 30 weeks old), and WT (7 weeks old) mice and their quantification (normalized to β-actin) relative to WT mice ($n = 3$ mice per group, biological replicates). Standard error depicted. Gender combined for statistical analysis. Data analyzed using one-way ANOVA with Tukey's multiple comparison test. Significant results: $^{**}p < 0.01$, $^{***}p < 0.001$, $^{****}p < 0.0001$. (C) Quantification of total ceramide (Cer), ceramide-1-phosphate (C1P), dihydroceramide (DHC), sphingosine (SPH), monohexosylceramide (MHC, including glucosylceramide and galactosylceramide), dihexosylceramides (CDH), monosialoganglioside 1 (GM1), and monosialoganglioside 3 (GM3) by flow injection electrospray ionization tandem mass spectrometry or liquid chromatography electrospray ionization tandem mass spectrometry in liver extracts from untreated P361R-FD mice (7 weeks old), HSC-transplanted P361R-FD mice (15 and 30 weeks old), and age-matched WT control mice ($n = 3$ mice per group, biological replicates). Standard error depicted. Gender combined for statistical analysis. Data analyzed using one-way ANOVA with Tukey's multiple comparison test. Significant results: $^{**}p < 0.01$; $^{***}p < 0.001$; $^{****}p < 0.0001$; ns, not significant. (D) Spleen sections from females of untreated P361R-FD mice (7 weeks old), HSC-transplanted P361R-FD mice (15 and 30 weeks old), and age-matched WT mice were stained using Masson trichrome or immunostained for macrophages (Galectin-3, Mac2) or lysosomes (CatD) (D). Scale bars, 250 μm.

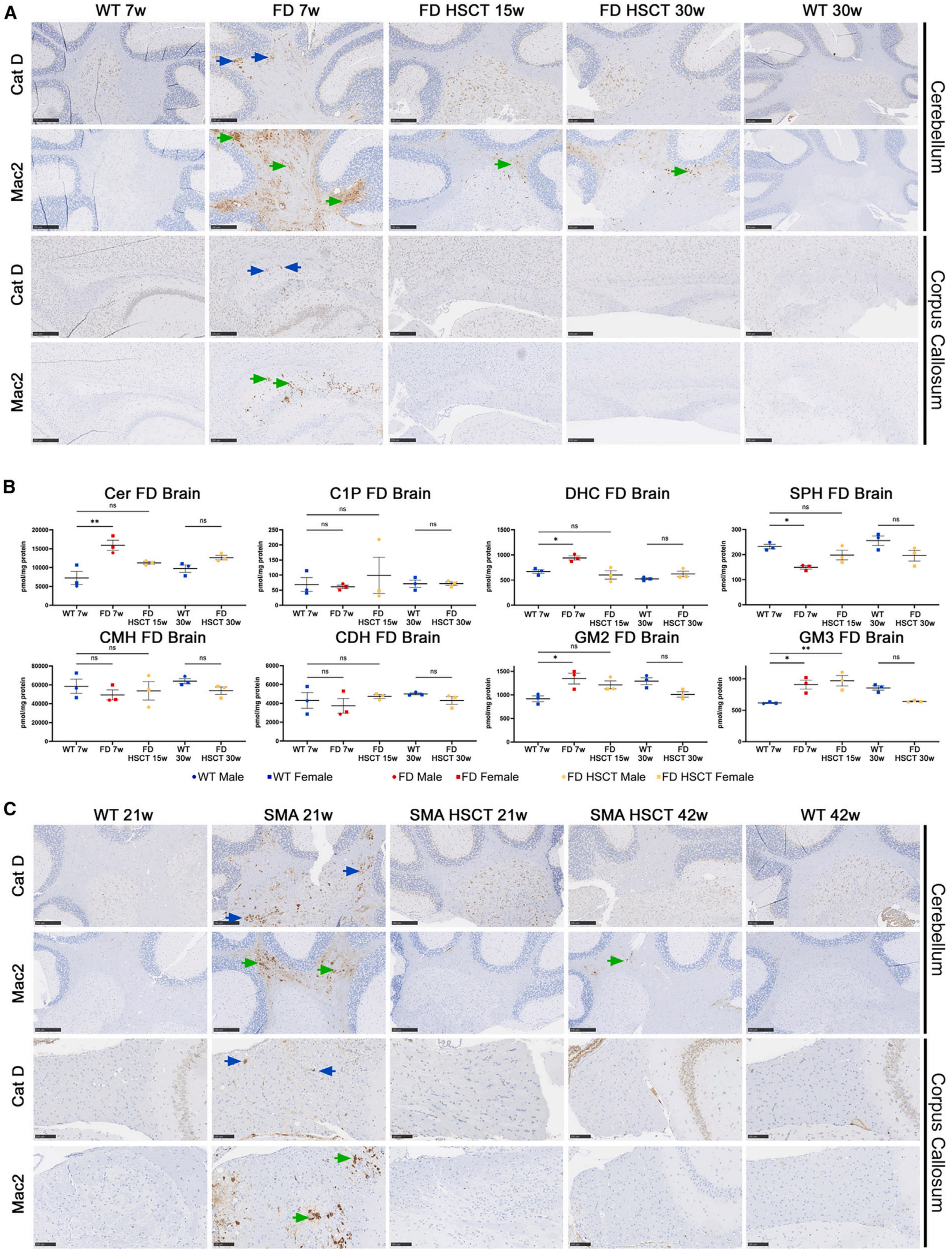

(legend on next page)

(Figure 3C). Total sphingosine (SPH), the product of Cer hydrolysis, remained normal in untreated P361R-FD (Figure 3C). Statistically significant increases in all of these studied sphingolipids noted in our untreated mutants was prevented following HSCT, through 30 weeks of age (Figure 3B). Mass spectrometry quantification of Cer species differing in acyl chain lengths revealed that all studied non-hydroxyl species (C16:0, C18:0, C20:0, C22:0, C24:0, C24:1, C24:2, C26:0, C26:1, C26:2) (Figure S3A) and almost all hydroxyl species (C18:0-OH, C22:1-OH, C24:1-OH, C24:2-OH, C26:0-OH, C26:1-OH, C22:0-OH) (Figure S3B) were found to be significantly elevated in untreated P361R-FD mice and normalized following HSCT at all time points studied. Cer(C24:0-OH) was the only Cer species still found elevated in 30-week-old P361R-FD following HSCT (Figure S3B).

Livers from untreated P361R-SMA mice were found to be atrophied (Figures S1C and S1D) in comparison to those from their WT age-matched littermates. HSCT resulted in improved liver weights at 21 weeks, but they were still found significantly atrophied by 42 weeks of age in P361R-SMA mice compared to age-matched WT mice. Interestingly, immunohistochemistry (IHC) examination confirmed very mild fibrosis (Masson trichrome, green arrows) as well as minimal CatD (blue arrows) and Mac2 accumulation (yellow arrows) in untreated P361R-SMA in comparison to P361R-FD mice (Figure S3C). These pathological findings were improved following HSCT, mainly at the younger age of 21 weeks compared to 42-week-old mice, as analyzed by IHC (Figure S3C). Those findings were subsequently quantified and confirmed by western blot analyses of liver lysates from untreated and treated P361R-SMA mice and their age-matched WT controls (Figure S3D). A significant reduction in phosphorylated STAT3 protein was seen in P361R-SMA following HSCT (Figure S3D).

Organomegaly is a common phenotype present in classical FD in patients and in the *Asah1*$^{P361R/P361R}$ mice.[25] Interestingly, while P361R-FD mice display an enlarged spleen (Figure S1B), spleen size in P361R-SMA mice was atrophied (normalized to length of the tibia) further confirming the two distinct phenotypes in our ACDase-deficient mice (Figure S1D). HSCT resulted in normalized spleen size and weight in both P361R-FD and P361R-SMA mice when first evaluated at 15 and 21 weeks of age, respectively. Interestingly, at the later time point analyzed, a possibly reduced spleen size and weight was observed in the HSCT mice, but it was found not to be statistically significant (Figure S1D). Advanced fibrosis (Masson trichrome, green arrows) along with massive accumulation of CatD+ lysosomal clusters (blue arrows) and increased infiltration of Mac2+ histiocytes (yellow arrows) in P361R-FD mice were prevented in HSCT-treated mice (Figure S3D). Only milder fibrosis was detected within the spleen parenchyma (red pulp) of HSCT-treated 30-week-old P361R-FD mice (Masson trichrome, green arrow) (Figure 3D). Similar to the liver, IHC examination of the spleen from P361R-SMA mice revealed a milder phenotype than was seen in P361R-FD mice. Observed pathology included the presence of large CatD+ lysosomal clusters (Figure S3E, blue arrow) and large Mac2+ accumulation (Figure S3E, yellow arrow) as well as mild fibrosis. Similar to spleens in treated P361R-FD mice, HSCT led to the prevention of these pathological findings (Figure S3E).

HSCT improves brain pathology

Neurologic involvement represents a critical part of the clinical phenotype of FD[29,30] with dominating cerebellar, cerebral cortex, and corpus callosum pathologies. Granuloma-like accumulations of abnormal CD68+ microglia/macrophages, as well as abnormal CatD-positive lysosomes and sphingolipid accumulation, were among the primary pathological findings previously described in two different mouse models of FD.[19,28] Here, we confirmed increased accumulation of CatD+ lysosomes (blue arrows) and increased Mac2+ macrophages (green arrows) in white matter of the cerebellum and corpus callosum in 7-week-old untreated FD-P361R mice (Figure 4A) that was almost prevented following HSCT in both 15- and 30-week-old mutant mice. Only minor increases in Mac2+ microglia/macrophages were observed in the cerebellum of P361R-FD mice following HSCT; but not near the extent observed in untreated mutant animals (Figure 4A). Following HSCT, the corpus callosum of treated P361R-FD mice was found to be free of storage pathology.

Mass spectrometry quantification of sphingolipids in brain extracts from 7-week-old P361R-FD mice confirmed significant accumulation of total Cer along with accumulation of DHC, GM2, and GM3 ganglioside in comparison with brain extracts from age-matched WT mice (Figure 4B). Surprisingly, all abnormal lipid accumulations found in the brains of untreated P361R-FD mice were prevented following HSCT, mainly at older ages in the case of GM2 and GM3 gangliosides (Figure 4B). Total Cer was also decreased to normal levels following HSCT (Figure 4B). Total SPH, decreased in untreated P361R-FD in comparison with WT, was also normalized in treated P361R-FD mice (Figure 4C). In contrast to the liver extracts from untreated P361R-FD, the other studied lipids such as C1P, CMH, and CDH were not significantly increased in untreated

Figure 4. Improved brain pathology in P361R-FD mice and P361R-SMA mice following HSCT

(A) Brain sections from female (cerebellum and corpus callosum) untreated P361R-FD mice (7 weeks old), HSC-transplanted P361R-FD mice (15 and 30 weeks old), and WT mice (7 and 30 weeks old) were immunostained for macrophages (Galectin-3, Mac2) or lysosomes (CatD). Scale bars, 250 μm. (B) Quantification of total Cer, C1P, DHC, SPH, MHC (including glucosylceramide and galactosylceramide), CDH, monosialoganglioside 2 (GM2), and GM3 by flow injection electrospray ionization tandem mass spectrometry or liquid chromatography electrospray ionization tandem mass spectrometry in brain extracts from untreated P361R-FD mice (7 weeks old), HSC-transplanted P361R-FD mice (15 and 30 weeks old), and age-matched WT control mice ($n = 3$ mice per group, biological replicates). Standard error depicted. Gender combined for statistical analysis. Data analyzed using one-way ANOVA with Tukey's multiple comparison test. Significant results: $^{*}p < 0.05$; $^{**}p < 0.01$,; ns, not significant. (C) Brain sections of female (cerebellum and corpus callosum) untreated P361R-SMA mice (21 weeks old), HSC-transplanted P361R-SMA mice (21 and 42 weeks old), and WT mice (21 and 42 weeks old) were immunostained for macrophages (Galectin-3, Mac2) or lysosomes (CatD). Scale bars, 250 μm.

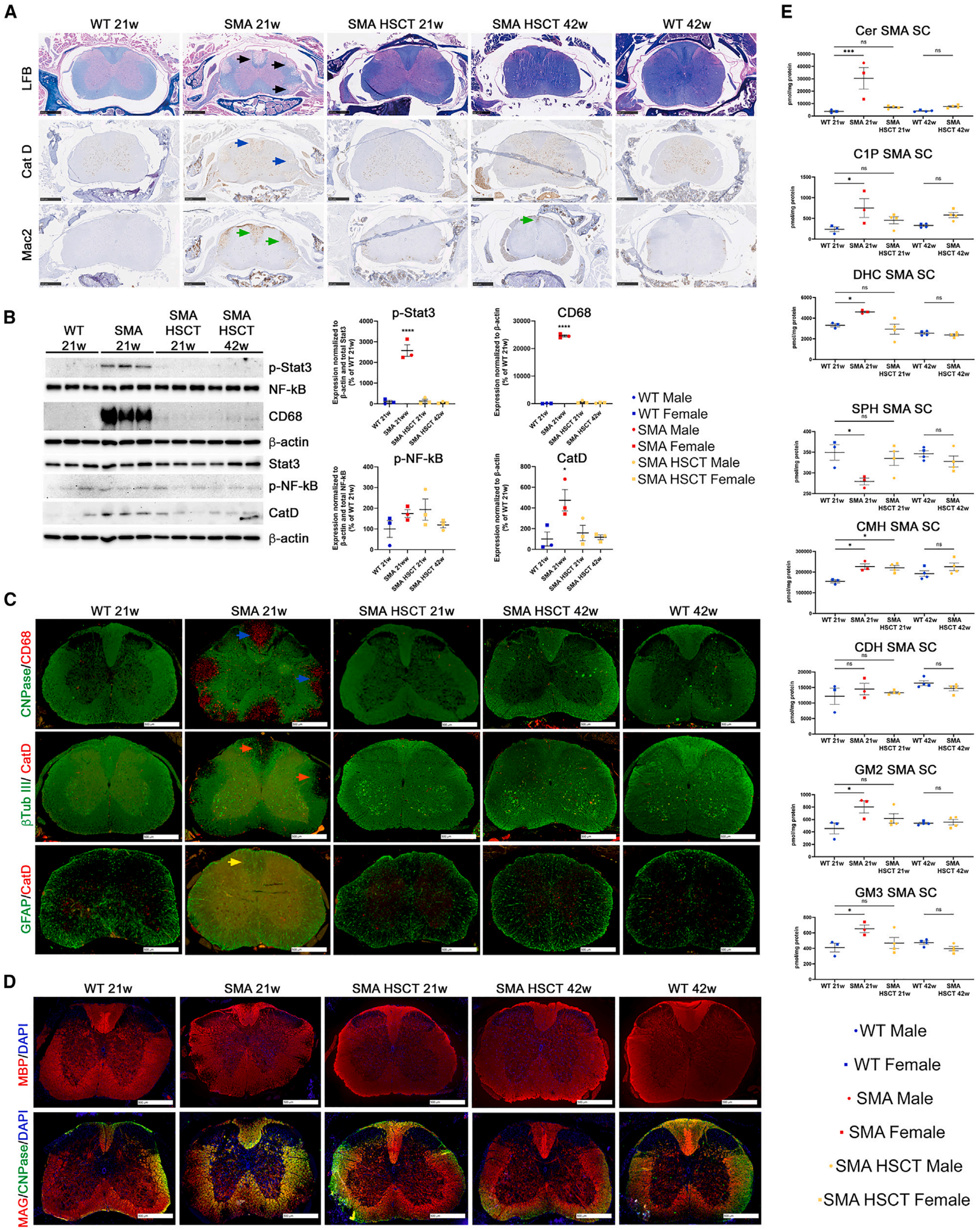

(legend on next page)

animals in comparison with WT and remained unchanged following HSCT (Figure 4B).

Similar to the liver, almost all studied non-hydroxy (Figure S4A) and hydroxy Cer species (Figure S4B) differing in acyl chain lengths were found significantly increased in brain extracts from untreated P361R-FD mice except for C24:0, C24:1, and C26:1, and hydroxy species C24:2-OH and C26:0-OH. HSCT in P361R-FD mice resulted in reduced accumulation of only certain non-hydroxy and hydroxy Cer species including 16:0, C18:0, C24:1, C24:2, C26:1, and C26:2 and hydroxyl species C18:0-OH, C24:1-OH, C24:2-OH, C26:0-OH, C26:1-OH, and C22:0-OH. Cer species C20:0, C22:0, C24:0, C26:0, C22:0-OH, C22:1-OH, and C24:0-OH were not normalized to control levels following HSCT in P361R-FD mice (Figures S4A and S4B). Those results contrast with the findings in the liver following HSCT, where only Cer C24:0-OH was found to be significantly elevated in comparison with age-matched WT following HSCT.

Previously, CD68+ staining for microglia and/or macrophages was found limited to the *arbor vitae* in the P361R-SMA cerebella, while the motor cortex did not display noticeable CD68+ staining.[26] Although total Cer and Cer species levels were increased in tissue lysates from P361R-SMA mice, changes were smaller than those previously reported in P361R-FD mice.[26] Here, we performed IHC on brain sections from untreated and treated P361R-SMA mice in comparison to WT control sections (Figure 4C). Similar to P361R-FD, we found increased accumulation of CatD+ lysosomes (Figure 4C, blue arrows) mainly in white matter of the cerebellum and the corpus callosum (Figure 4C, blue arrows) in comparison with age-matched WT. Although Mac2-positive microglia/macrophages (Figure 4C, green arrows) dominated the same brain regions, staining intensity was lower than that observed in P361R-FD mouse brains. Following HSCT, all treated animals were almost free of storage accumulation (Figure 4C). Similar to that found in treated P361R-FD mice, only trace amounts of increased Mac2+ microglia/macrophages were found in the cerebellum of the oldest SMA-P361R mice following HSCT.

Prevention of spinal cord pathology in P361R-SMA mice following HSCT

Profound and progressive demyelination, loss of axons, and altered sphingolipid levels in P361R-SMA spinal cords were the main pathological characteristics that we previously described in our P361R-SMA mice.[26] Here, we confirm massive demyelination and pronounced white-matter lesions throughout the dorsal, lateral, and ventral columns of the spinal cord in untreated P361R-SMA mice (Figure 5A, Luxol fast blue [LFB], black arrows) along with increased accumulation of CatD+ lysosomes (blue arrows) and massive infiltration of Mac2+ microglia/macrophages (green arrows) into the same regions. Surprisingly, all such pathological signs were normalized in all HSCT-treated P361R-SMA mice at 21 and 42 weeks of age. Minimal Mac2+ staining was noted in 42-week-old HSCT-treated P361R-SMA mice in comparison with untreated mice (Figure 5A).

Western blot quantification confirmed reduced CD68+ microglia/macrophage accumulation in treated animals along with normalization of CatD+ lysosomes to control levels following HSCT (Figure 5B). Increased accumulation of phosphorylated inflammatory proteins p-STAT3 and p-NF-κB was also noted in spinal cords from untreated P361R-SMA mice (Figure 5B). Similar to that observed in livers from P361R-FD mice, HSCT led to a decrease of both phosphorylated inflammatory proteins in treated P361R-SMA mice (Figure 5B).

Loss of oligodendrocytes (Figure 5C, reduced CNPase stain), increased CD68+ microglia/macrophage staining (Figure 5C, blue arrows), and decreased neuronal axon staining (Figure 5C, reduced βTub III stain, orange arrows) in the dorsal, lateral, and ventral spinal columns, along with reactivation of GFAP+ astrocytes with increased CatD+ staining (yellow arrows), were prominent in untreated P361R-SMA mice (Figure 5C).[26] Oligodendrocyte and axonal staining appeared improved following HSCT in the P361R-SMA mice. HSCT also prevented GFAP+ astrocyte reactivation/astrogliosis (Figure 5C). Immunofluorescence (IF) staining confirmed that reduced

Figure 5. Normalized spinal cord pathology in P361R-SMA mice following HSCT

(A) Spinal cord sections of females from untreated P361R-SMA mice (21 weeks old), HSC-transplanted P361R-SMA mice (21 and 42 weeks old), and WT (21 and 42 weeks old) mice stained using Luxol fast blue (LFB) or immunostained for macrophages (Mac2, Galectin-3) or lysosomes (CatD) (A). Scale bars, 500 μm. (B) Representative immunoblots of CatD, CD68, and total and phosphorylated inflammatory proteins STAT3 and NF-κB in spinal cord lysates from untreated P361R-SMA mice (21 weeks old), HSC-transplanted P361R-SMA (21 and 42 weeks old), and WT (21 weeks old) mice along with quantification (normalized to β-actin) relative to WT mice ($n = 3$ mice per group, biological replicates). Standard error depicted. Gender combined for statistical analysis. Data analyzed using one-way ANOVA with Tukey's multiple comparison test. Significant results: $^*p < 0.05$, $^{****}p < 0.0001$. (C) Spinal cord sections of females from untreated P361R-SMA mice (21 weeks old), HSC-transplanted P361R-SMA mice (21 and 42 weeks old), and WT control (21 and 42 weeks old) mice were used for immunofluorescence staining to detect changes in neural cell representation: CNPase-positive astrocytes (CNPase), beta-tubulin III-positive neurons (βTubIII), and glial fibrillary acidic protein (GFAP)-positive astrocytes. Sections were also stained for CatD-positive lysosomes (CatD) and CD68+ macrophages (CD68). Scale bars, 500 μm. (D) Spinal cord sections of females from untreated P361R-SMA mice (21 weeks old), HSC-transplanted P361R-SMA mice (21 and 42 weeks old), and WT control mice (21 and 42 weeks old) were used for immunofluorescence staining of myelination proteins: myelin basic protein (MBP), myelin-associated glycoprotein (MAG), as well as CNPase positive astrocytes (CNPase) and DAPI nuclear staining. Scale bars, 500 μm. (E) Quantification of total Cer, C1P, DHC, SPH, MHC (including glucosylceramide and galactosylceramide), CDH, GM2, and GM3 by flow injection electrospray ionization tandem mass spectrometry or liquid chromatography electrospray ionization tandem mass spectrometry in spinal cord extracts from untreated P361R-SMA mice (21 weeks old), HSC-transplanted P361R-SMA mice (21 and 42 weeks old), and WT control mice (21 and 42 weeks old) ($n = 3$–4 mice per group, biological replicates). Standard error depicted. Gender combined for statistical analysis. Data analyzed using one-way ANOVA with Tukey's multiple comparison test. Significant results: $^*p < 0.05$; $^{**}p < 0.01$; $^{***}p < 0.001$; ns, not significant.

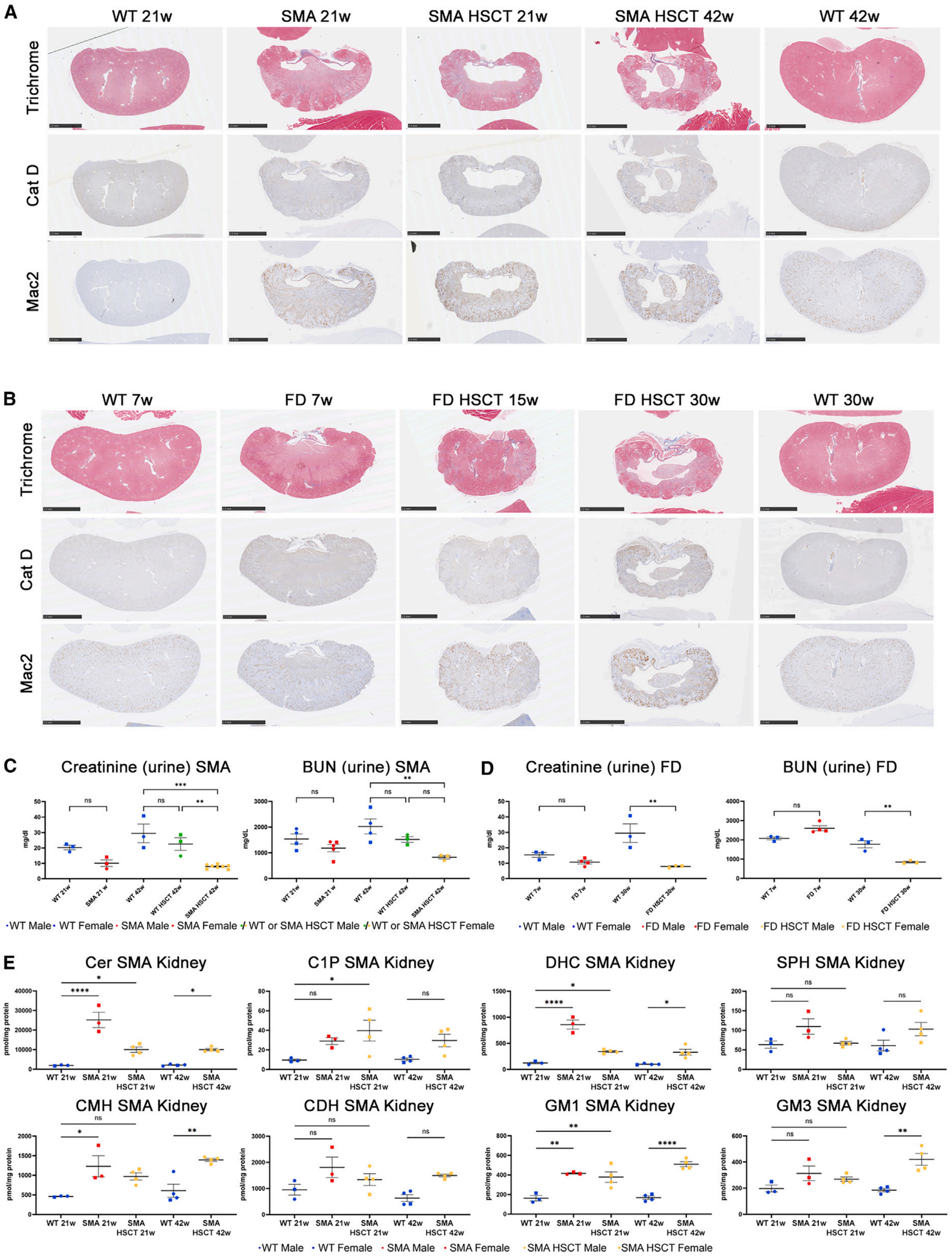

(legend on next page)

myelination proteins, including myelin-associated glycoprotein (MAG) and myelin basic protein (MBP), correlated with decreased CNPase staining throughout the dorsal, lateral, and ventral columns of spinal cord in untreated P361R-SMA mice (Figure 5D). HSCT treatment prevented loss of MAG and MBP in the spinal cord of P361R-SMA mice.

Quantification of sphingolipids by mass spectrometry in the spinal cord extracts from untreated 21-week-old P361R-SMA mice confirmed massive accumulation of total Cer along with profound accumulation of C1P, DHC, CMH, GM2, and GM3 gangliosides in comparison with age-matched WT mice (Figure 5E). CDH remained in the normal range. Total SPH was significantly decreased in untreated P361R-SMA mice in comparison with WT mice (Figure 5E). Interestingly, pathological lipid accumulation was almost completely prevented in all P361R-SMA mice at the age of 21 and 42 weeks following HSCT (Figure 5E). All studied non-hydroxy and hydroxy Cer species differing in acyl chain lengths were found to be significantly increased in spinal cord extracts from untreated P361R-SMA mice (Figures S5A and S5B). HSCT resulted in prevention of almost all abnormal accumulation of studied Cer species except C26:0, which, while trending toward a reduction, was still found to be statistically elevated (Figures S5A and S5B).

The spinal cords were not as affected in P361R-FD as in P361R-SMA mice (Figure 5C). LFB staining did not reveal areas of significant demyelination in the spinal cords from untreated P361R-FD mice, but these were easily seen in P361R-SMA mice (Figures S4C and 5A). Only minor accumulation of CatD+ lysosomes (blue arrows) and Mac+ microglia/macrophages (green arrows) were seen in the dorsal and lateral spinal cord regions in untreated P361R-FD mice (Figure S5C). Results were confirmed by western blot quantification from spinal cord lysates (Figure S5D). Only modestly increased p-STAT3, CatD, and CD68 levels were seen in 7-week-old untreated P361R-FD mice, and this increase was not seen in 15-week-old mutants following HSCT. Only 30-week-old P361R-FD mice showed pronounced IHC Mac2 staining (Figure S5C) following HSCT, and this correlated with quantification of CD68 western blots (Figure S5D, green arrows).

Kidney failure as a potential cause of premature death in both P361R-FD and P361R-SMA mice

Despite a previous report of increased Cer in the kidneys of *Asah1*$^{P361R/P361R}$ mice,[25] and elevated blood urea nitrogen (BUN) in *Asah1*^{tmEx1} mice,[28] a detailed kidney examination has not been completed in ACDase-deficient mouse models or patients. Interestingly, the kidney showed obvious impairment following HSCT in both P361R-FD mice and P361R-SMA mice at a young age (Figures S1A–S1D). The kidneys from 21-week-old P361R-SMA mice that underwent HSCT were pale in color and smaller in size and weight in comparison to those from both untreated P361R-SMA mice and WT mice (Figures S1C and S1D). This phenotype worsened with age. IHC examination revealed that the tremendous fibrosis seen in untreated P361R-SMA mouse kidneys was not improved following the HSCT treatment (Figure 6A). Although the kidneys from HSC-transplanted P361R-SMA mice showed an improvement in accumulated lysosomal CatD at 21 week of age, the massive infiltration of Mac+ macrophages was even worse in HSCT-treated P361R-SMA mice in comparison with untreated SMA mice of the same age (Figure 6A). Forty-two-week-old P361R-SMA mice, following HSCT, showed obvious deterioration in kidney size and overall disruption of normal kidney morphology accompanied by macrophage infiltration and lysosomal accumulation in comparison to WT mice (Figure 6A).

Even though 7-week-old untreated P361R-FD mice did not show pronounced fibrosis as seen in untreated P361R-SMA mice, they exhibited increased CatD and Mac2 accumulation in comparison with age-matched WT mice (Figure 6B). Like P361R-SMA mice, HSC-treated P361R-FD mice showed a slight improvement in CatD+ lysosomal accumulation at 15 weeks of age, but they also started to exhibit pronounced fibrosis and increased infiltration of Mac+ macrophages not seen in untreated mutant animals (Figure 6B). By 30 weeks of age, HSCT-treated P361R-FD mice showed more pronounced deterioration of all studied pathological characteristics in comparison to P361R-SMA mice (Figure 6B).

Creatinine and BUN are widely used to evaluate kidney function/glomerular filtration rate. In kidney disease, creatinine levels in the

Figure 6. Kidney pathology in both P361R-FD mice and P361R-SMA mice following HSCT

(A) Kidney sections of females from untreated P361R-SMA mice (21 weeks old), HSC-transplanted P361R-SMA mice (21 and 42 weeks old), and WT control mice (21 and 42 weeks old) were stained using Masson trichrome or immunostained for macrophages (Galectin-3, Mac2) or lysosomes (CatD) (A). Scale bars, 2.5 mm. (B) Kidney sections of females from untreated P361R-FD mice (7 weeks old), HSC-transplanted P361R-FD mice (15 and 30 weeks old), and WT mice (7 and 30 weeks old) were stained using Masson trichrome or immunostained for macrophages (Galectin-3, Mac2) or lysosomes (CatD) (B). Scale bars, 2.5 mm. (C) Urine collected from untreated P361R-SMA mice (21 weeks old, red), HSC-transplanted P361R-SMA mice (42 weeks old, orange), WT mice (21 and 42 weeks old, blue), and HSC-transplanted WT mice (42 weeks old, green) was used for detection of creatinine and blood urea nitrogen (BUN) (n = 3–5 mice per group, biological replicates). Standard error depicted. Gender combined for statistical analysis. Data analyzed using one-way ANOVA with Tukey's multiple comparison test. Significant results: $^{**}p < 0.01$; $^{***}p < 0.001$; ns, not significant. (D) Urine collected from untreated P361R-FD mice (7 weeks old, red), HSC-transplanted P361R-FD mice (30 weeks old, orange), and WT mice (7 and 30 weeks old, blue) was analyzed for creatinine and BUN (n = 3–5 mice per group, biological replicates). Standard error depicted. Gender combined for statistical analysis. Data analyzed using one-way ANOVA with Tukey's multiple comparison test. Significant results: $^{**}p < 0.01$; ns, not significant. (E) Quantification of total Cer, C1P, DHC, SPH, MHC (including glucosylceramide and galactosylceramide), CDH, GM1, and GM3 by flow injection electrospray ionization tandem mass spectrometry or liquid chromatography electrospray ionization tandem mass spectrometry in kidney extracts from untreated P361R-SMA mice (21 weeks old), HSC-transplanted P361R-SMA mice (21 and 42 weeks old), and WT mice (21 and 42 weeks old) (n = 3–4 mice per group, biological replicates). Standard error depicted. Gender combined for statistical analysis. Data analyzed using one-way ANOVA with Tukey's multiple comparison test. Significant results: $^{*}p < 0.05$; $^{**}p < 0.01$; $^{****}p < 0.0001$; ns, not significant.

blood are elevated, whereas the creatinine clearance rate and urine levels are diminished (as well as a decreased level of BUN). We tested both creatinine level and BUN in both P361R-FD mice and P361R-SMA mice with and without HSCT treatment in comparison to age-matched WT control mice (Figures 6C and 6D). Untreated P361R-SMA mice showed slight increases in both creatinine and BUN in comparison with WT mice; this became more obvious and statistically significant following HSCT at 42 weeks of age in P361R-SMA mice (Figure 6C). Because kidney disease/acute kidney injury is known to be a common complication following HSCT,[28,31,32] we also assayed creatinine and BUN in 42-week-old WT mice following HSCT to see if the observed kidney pathology results from HSCT itself (Figure 6C). In these HSC-treated WT mice creatinine and BUN were slightly decreased in comparison with untreated WT mice, but not statistically significant as seen in HSC-treated P361R-SMA mice (Figure 6C, light blue). Levels of creatinine and BUN in urine from untreated P361R-FD mice were similar to those in controls, but significantly decreased in P361R-FD mice following HSCT at 30 weeks of age, consistent with the results seen in HSC-treated P361R-SMA mice (Figure 6D).

Because untreated P361R-SMA mice exhibited more profound kidney pathology in comparison with untreated P361R-FD mice (advanced fibrosis and massive CatD accumulation), we decided to quantify lipids of kidney extracts from P361R-SMA mice with and without HSCT by mass spectrometry (Figure 6E). We noted increased accumulation of total Cer, C1P, DHC, CMH, GM1, and GM3 in untreated P361R-SMA mice in comparison with controls. HSCT led to significant decreases in total Cer, C1P, and DHC in lysates from P361R-SMA mice at both 21 and 42 weeks of age, but the reductions were generally not to normal WT levels (Figure 6E). Interestingly, CMH, GM1, and GM3 accumulation was even more profound in 42-week-old HSC-treated P361R-SMA mice in comparison with untreated 21-week-old P361R-SMA mice. SPH was not significantly increased in untreated animals in comparison with WT mice and remained unchanged following HSCT (Figure 6E).

As in the spinal cord, almost all studied non-hydroxy and hydroxy Cer species differing in acyl chain lengths were significantly increased in kidney extracts from untreated P361R-SMA mice, except for C26:0-OH (Figures S6A and S6B). HSCT treatment of P361R-SMA mice prevented abnormal accumulation of certain non-hydroxy and Cer species only including C16:0, C18:0, C24:0, C24:1, C24:2, C26:0, C26:1, and C26:2 (Figure S6A) and hydroxy species C26:1-OH (Figure S6B). Cer species C20:0, C22:0, and almost all hydroxy species including C18:0-OH, C22:0-OH, C22:1-OH, C24:0-OH, C24:1-OH, and C24:2-OH were improved in HSC-treated 21-week-old P361R-SMA mice more so than in 42-week-old P361R-SMA mice, but they were not normalized to WT levels.

DISCUSSION

We have previously generated and thoroughly characterized mouse models of both FD and SMA-PME-like disorders that demonstrate similar phenotypes to those seen in patients.[19–21,23–26] In this study, we evaluated HSCT in both models of ACDase deficiency. This provides a unique opportunity to compare the effects of HSCT in two distinct phenotypes with pathological involvement in different organ systems. Given that earlier initiation of HSCT is predicted to lead to better outcomes (as irreversible organ and tissue damage has less time to occur), we used 4-week-old P361R-FD mice, a time point where only milder pathology has been reported,[19] as our early time point for administering treatment. Untreated P361R-FD mice usually die around 7 weeks of age. We began treatment in P361R-SMA mice at the age of 6–8 weeks when no brain pathology has been detected.[26] Encouragingly, both sets of treated mice displayed improved lifespans, 4.4 times in the case of P361R-FD and three times in P361R-SMA mice. Both mutant genotypes remained smaller in size than their WT control littermates following HSCT, an often-described side effect following HSCT in LSD mice.[33]

Hematopoietic dysfunction is common in FD patients and FD mouse models as shown by splenomegaly, lymphocytosis, neutrophilia, anemia, as well as excess cytokine release in plasma (MCP-1, IL-6) and macrophage infiltration into many organs.[1,22,34,35] CBC analysis revealed overall improvement of all altered hematopoietic characteristics in both P361R-FD mice and P361R-SMA mice including prevention of lymphocytosis and neutrophilia in both models following HSCT at all studied ages. Iron-deficiency anemia, more pronounced in P361R-FD than in P361R-SMA mice, was prevented in younger animals at the age of 15 weeks but gradually appeared in treated P361R-FD animals by 30 weeks of age (Figure 2C). Significant infiltration of large foamy macrophages contributing to splenomegaly was apparent in P361R-FD mice, while spleens from P361R-SMA mice were atrophied but contained fewer accumulated macrophage cells. These pathological characteristics were improved following HSCT (Figures 3D and S3E).

MCP-1 is a cytokine that attracts immune cells to sites of inflammation and is important for macrophage recruitment.[36] Elevated MCP-1 is well known in ACDase deficiency and has been discussed as a biomarker of FD and its response to treatment.[1] We evaluated cytokine levels every 7 weeks after HSCT in both disease phenotypes. Surprisingly, we did not see an increase in MCP-1 at any time point. These results suggest that providing a more normal hematopoietic environment greatly modifies MCP-1 release and confirm that modulation of the hematopoietic system can have an important role in disease progression.

While ERT strategies are used to treat several LSDs, to date ERT remains largely unsuccessful for patients with neurological involvement such as in FD.[37] In this context, HSCT may have an advantage over ERT as donor-derived cells migrate across the BBB and can differentiate into CNS macrophages and microglia, which may secrete the corrective enzyme into the CNS, and improve neurocognitive outcomes.[38] HSC may thus be a superior method for the treatment of ACDase deficiency due to its potential to address prominent neurological involvement. The correction of CNS manifestations by HSCT has been reported in other neuropathic LSDs, with better

treatment outcomes in some, but not all, LSDs if this procedure was performed prior to the development of neurological symptoms.[39] Because neurological involvement is critical in both FD and SMA-PME, efforts to treat ACDase-deficient patients by HSCT have been carried out in several cases. It has been reported that BM transplants may improve granulomas in FD patients with little or no lung or nervous system complications[40,41] as well as considerable improvements in the number of subcutaneous nodules, mobility, and pain in FD patients without CNS involvement.[3,15,18,34,42] This has not been evaluated in SMA-PME patients yet.[4] In our mouse models, treatment of P361R-FD was initiated at the age of 4 weeks wherein only milder pathology has been reported, and P361R-SMA mice were treated at the age of 6–8 weeks when no brain pathology was detected.[26] Neither mouse model exhibited any decline in behavioral/functional characteristics in the open-field, Rotarod, or wire hang test at these young ages prior to treatment initiation. These behavioral tests confirmed a huge decline in all studied behavioral performances at their disease end state. Brain pathology in ACDase deficiency was dominated by microglia/macrophage infiltration along with increased accumulation of clustered lysosomes in white matter of the cerebellum and corpus callosum at disease end-stage in both P361R-FD and P361R-SMA mice. Almost all these pathological findings, along with altered expression of total Cer and its metabolites, were improved following HSCT. We also observed improvements in all behavioral tests in comparison with untreated mice close to end-stage time points. These results suggest early transplantation for successful correction of function as well as biochemical readouts in these ACDase-deficiency disorders.

Spinal cord demyelination and neuronal loss dominate both SMA-PME patients and our SMA-PME-like mouse model.[5,26] Little preclinical work has investigated treatments for SMA-PME, and the impact of HSCT on spinal cord pathology in SMA-PME has not yet been evaluated, but some studies have suggested its potential. Adding recombinant human ACDase to SMA-PME patient-derived dermal fibroblasts significantly reduced total Cer abundance.[43] Considering SMA type 1, allogeneic adipose-derived mesenchymal stem cell therapy may yield promising results for combating the neurodegeneration of alpha motor neurons in the spinal cord.[44] Moreover, adult HSC transplanted into the spinal cords of a mouse model of motor neuron degeneration had a neuroprotective effect.[45] In our study, IHC analysis revealed an impressive prevention of demyelination, microglia/macrophage infiltration, pathological accumulation of lysosomes, loss of neurons and oligodendrocytes, and loss of myelination proteins MBP and MOG in spinal cord sections from P361R-SMA mice following HSCT. Mass spectrometry analyses revealed further clearance of the pathologically altered sphingolipid accumulation dominated by a reduction in total Cer and its species in spinal cord extracts of P361R-SMA mice following HSCT. We also found reduction of elevated C1P, which represents an important signal lipid and may point to pathological conditions by supporting pro-proliferative signaling. This was also corrected by HSC therapy. Following HSCT, not even 42-week-old P361R-SMA mice demonstrated the same degree of spinal cord pathology seen in untreated animals. Our results suggest a positive outcome of HSC treatment in SMA-PME mice when introduced early and when only mild pathology exists in these animals. Interestingly, untreated P361R-FD mice did not show such spinal cord pathology, further confirming distinct phenotypes in our two models of ACDase deficiency.

The kidney was the only organ studied that showed an obvious negative impact due to HSCT, and this was noted in both models. Even though both mutant mice showed improvement in pathological accumulation of CatD when first tested at an earlier age (15 and 21 weeks for P361R-FD and P361R-SMA, respectively) following HSCT, the kidneys were pale and advanced fibrosis along with macrophage infiltration was detected, which continued to worsen as the animals aged. Moreover, decreased urea nitrogen and creatinine levels for glomerular filtration rate showed impairment only in ACDase-deficient mice following HSCT in comparison with untreated animals and controls. Severe proteinuria and albuminuria along with increased accumulation of Cer and signaling C1P were found in mice with *Asah1* podocyte-specific gene deletion (*Asah1*$^{fl/fl}$/Podo^{Cre} mice), suggesting an essential role of ACDase for the maintenance of the structural and functional integrity of podocytes.[46] However, initial improvement in lysosomal CatD accumulation along with decreased total Cer accumulation and C1P levels suggests that worsening kidney pathology could be due to irradiation and/or HSC transplantation rather than ACDase deficiency itself. Kidney injury after HSCT has been described as a common complication and can contribute to morbidity and mortality.[47,48] Moreover, transplant-related organ toxicity occurs after allogeneic HSCT in 16.5% of patients with acute leukemia due to kidney (7.3%), multi-organ (6.0%), and liver toxicity (2.6%).[49] We observed ganglioside GM1 and GM3 being the main lipids to display pathological accumulation in P361R-SMA mice following HSCT, while total Cer and C1P levels improved over time following treatment. Abundant GM3 has previously been found in the foot processes of podocytes maintaining functional integrity of the filtration barrier and thus a contributor to podocyte injury in diabetes-associated nephropathy.[50] GM1 has been associated with cell-cycle arrest and hypertrophy of renal mesangial cells of the glomerulus.[50,51] Gangliosides such as these have an important role in the maintenance of glomerular homeostasis and thus can be associated with impairment of glomerular filtration rate in our models of ACDase following HSCT. Creatinine and BUN were slightly decreased in HSC-treated WT mice in comparison to untreated WT mice, but not to a statistically significant degree as seen in HSC-treated P361R-SMA mice. These results suggest that irradiation itself does not have a profound negative impact on glomerular homeostasis or sphingolipid alteration in mice following treatment. Whether irradiation or HSCT contributes to worsening pathological alteration of gangliosides and resultant kidney impairment in ACDase-deficient conditions remains unclear. Our results here suggest taking a particularly close look at the kidney while treating LSD patients with HSCT.

Clinical implementation of HSCT has been rare in FD patients, and no gene-therapy studies have yet been launched to treat FD. Neither

HSCT nor gene therapy has been attempted in SMA-PME patients as the lack of a proper SMA-PME mouse model to date has left the benefit of such treatment unknown. Here, we show that HSCT has proved to be an effective therapeutic option for both SMA-PME and FD. Although the kidney showed deterioration following HSCT, the functional improvements and benefits seen in other symptoms that are hallmarks for ACDase deficiencies, along with a significantly increased survival rate, makes HSCT an interesting treatment option for those with FD or SMA-PME. It is also possible that autologous HSCT could be further enhanced using gene therapy to facilitate a supranormal degree of enzyme production. Such a strategy would transduce a patient's own HSCs by way of a viral vector *ex vivo* and infuse modified cells back into the patient following appropriate conditioning to obviate risk of graft-versus-host diseases. Such a strategy may have promise for the treatment of ACDase deficiencies.

MATERIALS AND METHODS

Mice

P361R-FD and P361R-SMA mice were generated as described previously.[25,26] These mice were backcrossed for more than six generations to C57BL/6J mice purchased from Jackson Labs. Genome SNP scanning was contracted to The Jackson Laboratory, and the mice were confirmed to be near isogenic to C57BL/6J.[26] To generate homozygous P361R-FD, P361R-SMA, and control mice, we crossed heterozygotes and genotyped as previously reported.[25] Mice were housed in pathogen-free conditions. All animal procedures were approved and carried out in strict adherence to the policies of the Animal Care Committee and the Medical College of Wisconsin (MCW) Institutional Animal Care and Use Committee (IACUC).

BM isolation and transplantation

Total BM was isolated from the tibia, femur, and ilium of 6- to 9-week-old WT mice as described previously.[52] Briefly, the mice were euthanized with CO_2 and sprayed with 70% ethanol. Tibia, femur, and ilium of lower limbs were cut off and transferred into a flushing buffer (3% FBS, 0.2mM EDTA in PBS) on ice. In a biological safety cabinet, a 27G $\times$ 0.5 in. needle (305109, BD Biosciences Canada, Mississauga, Canada) and a 20-mL syringe filled with ice-cold flushing buffer were used to flush the BM cells onto a 70-μm nylon cell strainer placed in a 50-mL Falcon conical tube. BM cells were centrifuged at 500 $\times$ *g* for 7 min at 4°C and supernatant was removed. A Lineage Cell Depletion Kit (Miltenyi Biotec, Bergisch Gladbach, Germany) was used for purification of HSCs in accordance with the manufacturer's instruction. P361R-FD mice were whole-body irradiated with 9 Gy at 4 weeks of age, while P361R-SMA mice were irradiated with 11 Gy at 6–7 weeks of age. One million live cells were injected retro-orbitally into all recipients.

Survival and weight data

Mouse survival was recorded for Kaplan-Meier curves. Whole-body weight was recorded weekly. For organ analyses, mice of desired ages were euthanized with CO_2 gas, weighed, and photographed using the Xiaomi Mi Note phone camera. Organ weights were normalized to whole-body weight or tibia length.

Peripheral blood and serum biochemistry, cytokine analysis by ELISA

Peripheral blood was collected from the facial vein or by cardiac puncture into EDTA-coated microtainers (BD Biosciences Canada, Mississauga, Canada). Afterward, 20 μL were taken from the blood sample and added to 120 μL of CELLPACK DCL (Sysmex #DCL-300A, Kobe, Japan) diluent. Samples were processed on a Sysmex XN-1000 Hematology Analyzer (Kobe, Japan) using the Pre-Dilution setting. Results were calculated by the Sysmex XN-1000V-1-A software (Kobe, Japan). For plasma analysis, blood samples were centrifuged at 1,000 $\times$ *g* for 10 min and the plasma was immediately collected and stored at −80°C until use. Mouse IL-6 (catalog no. [cn]: 431304, BioLegend, San Diego, CA) and mouse monocyte chemotactic protein-1 (MCP-1; cn: 432701, BioLegend, San Diego, CA) ELISA kits were used to detect the concentration of cytokines in plasma in accordance with the manufacturer's instructions. All samples were blinded to the performer of the analysis.

Histopathology and IHC

Mice were euthanized by CO_2 inhalation. Selected organs were collected and immediately fixed in 10% phosphate-buffered formalin for 24–48 h. Organs were subsequently embedded in paraffin and sectioned at 4 μm. Dissected spinal cords were decalcified with Immunocal Decalcifier (141432 StatLab, McKinney, TX, USA) for 3 h prior to embedding and sectioning. Spleen, liver, and kidney sections were stained with hematoxylin and eosin (H&E, 7211, McKesson Medical-Surgical, Evanston, IL) along with Masson trichrome stains (Newcomer Supply, Middleton, WI). Brain and spinal cord sections were stained with H&E and LFB (1328-51-4, Sigma-Aldrich). IHC was performed with the following primary antibodies: rat anti-mouse Mac2 (1:500, Galectin-3 clone M3/38, Cedarlane, Burlington, NC) and rabbit CatD (1:5,000, clone EPR3057Y, ab75852, Abcam Cambridge, MA). The following secondary antibodies and kits were used to detect primary antibodies: biotinylated rabbit anti-rat Immunoglobulin G (IgG) (1:400, BA-9401, Vector laboratories, Burlingame, CA), biotinylated goat anti-rabbit IgG (1:500, 711-066-152,Vector Laboratories), and Betazoid DAB kit Chromogen (BDB2004, Biocare Medical, Pacheco, CA) according to the manufacturer's instructions. Slides were scanned on the Aperio AT2 histology slide scanner (Leica Biosystems, Buffalo Grove, IL) or NanoZoomer 2.0-HT histology slide scanner (Hamamatsu Photonics, Ichinocho, Japan). Scanned micrographs were analyzed with Aperio ImageScope analysis software (Leica Biosystems, Grove, IL). Most of the samples were blinded to the performer of the analysis.

IF analysis

Spinal cord sections (as prepared above) were also used for IF analysis. Tissue sections were deparaffinized with two xylene washes, one xylene:ethanol (1:1) wash, one 100% ethanol wash, one 95% ethanol wash, one 70% ethanol wash, one 50% ethanol wash, and two washes in distilled water (3 min each), all at room temperature.

Antigen retrieval was carried out by heating sections at 95°C in citrate buffer at pH 6.0 (C9999, Sigma-Aldrich) for 30 min. Sections were then incubated with rodent block M (RBM961, Biocare Medical, Pacecho, CA) for mouse-on-mouse staining, and washed sections were then blocked in 5% bovine serum albumin (BSA) in PBS. The following primary antibodies were used: CatD (1:1,000, clone EPR3057Y, ab75852, Abcam), CD68 (1:400, 97778S, Cell Signaling), CNPase (1:400, clone CL2887, NBP2-46617, Novus Biological), βIII-tubulin (1:400, clone TuJ, MAB1195SP, R&D Systems), GFAP (1:200, clone GA5, 3670T), MBP (1:400, 78896S), and MAG (1:400, 9043T, all from Cell Signaling) with overnight incubations. Sections were then washed with PBS and labeled with fluorescence-conjugated secondary antibodies: Alexa Fluor 488 goat anti-mouse (1:1,000, A11001, Thermo Scientific) and Alexa Fluor 594 goat anti-rabbit (two droplets per mL, R37117, Thermo Scientific) for 1 h at 37°C. The cells were then washed with PBS (5 × 5 min) and mounted using Vectashield mounting medium (H-2000, Vector Laboratories, Burlingame, CA) containing 4′,6-diamidino-2-phenylindole (DAPI) to counterstain cell nuclei. Whole slides were scanned at high resolution on a VS120 slide scanner (Olympus) using a Plan-APO 20× lens (located at the CRI Imaging core, MCW). Images were viewed, and background and brightness adjusted using OlyVIA software v3.2.1 (Olympus). Regions of interest were exported in the TIFF format, and images were cropped and post-processed using paint.net v4.2.15 (dotPDN). Most of the samples were blinded to the performer of the analysis.

Behavioral studies

Motor coordination in P361R-SMA mice and WT mice was tested by Rotarod assays (IITC Life Science, Woodland Hills, CA). The mice were placed on the device and subjected to acceleration from 4 to 40 rpm for a total of 5 min. The time until a mouse fell off, reported as latency, was automatically recorded. Animals were subjected to three trials per day for two consecutive days. To test muscle strength of both P361R-SMA mice and P361R-FD mice, the animals were placed on a metal mesh surface (inverted screen frame: 12″ long by 12″ wide by 1″ high) that was enclosed by smooth metal walls. The surface was slowly turned upside down, allowing the mice to hold on. The time to fall, reported as hang time, was recorded to a maximum of 1 min. Animals were subjected to three trials per day for two consecutive days. All tests were performed between 8 am and 12 pm. To reduce non-study-related variability, all tests were performed with the same apparatus in the same closed room. Experimenter variability was reduced by having the same person perform each trial of a given test on all animals. Voluntary locomotor and exploratory activities along with assessments of anxiety-like behavior in P361R-FD mice were tested in an open-field arena. The mice were placed into a large circular cylinder (diameter 19″, height 12.5″). An automatic tracking system (ANY-Maze Tracking Software, Stoelting, Chicago, IL, USA) was used to record the total distance traveled, time mobile, time freezing, mean speed, the number of entries into the inner zone of the arena, as well as time mobile, total time, and time freezing in the inner zone. The number of entries into the inner zone was used as a measure of thigmotaxis, a tendency for mice to stay close to the walls of the testing apparatus due to anxiety induced by the novel environment. Videos were recorded using a SONY Handycam camera over a 10-min period per trial. All tests were performed between 8 am and 12 pm. As above, to reduce non-study-related variability, all tests were performed with the same apparatus in the same closed room. Due to body size differences of mutants and WT mice and effect of irradiation, the performer of the behavioral studies could not be blinded to the mice.

Western blotting

Frozen livers and spinal cords were homogenized in RIPA buffer (89900, Thermo Scientific) containing HALT Protease (78429, Thermo Scientific) and Phosphatase Inhibitor Cocktail (815-968-0747, Thermo Scientific) using a Bullet Blender Storm 24 tissue homogenizer (Next Advance) with 0.5-mm zirconium oxide beads. Homogenates were clarified by centrifugation (19,000 × g for 10 min at 4°C) and lysates were then used for protein concentration determination using a bicinchoninic acid (BCA) assay kit (Thermo Fisher Scientific). An equal amount of protein (20 μg) for each sample was separated by SDS-PAGE and subsequently transferred to a polyvinylidene fluoride (PVDF) membrane (Millipore, Billerica, MA). Membranes were blocked with 5% milk powder for 1 h at RT. The following primary antibodies were then used: CatD (2:10,000, ab75852, Abcam), STAT3 (4904), p-STAT3 (9145), NF-κB (8242), p-NF-κB (3033), CD68 (97778S), and albumin (4929S) LC3A/B (12741S all from Cell Signaling Technologies and at 1:1,000). All primary antibodies were diluted in 5% BSA in TAE buffer (50× solution: 484 g of Tris base, 77-86-1 Sigma-Aldrich; 200 mL of 0.5 M EDTA, AM9260G, Thermo Fisher Scientific; 114.2 mL of acetic acid, 695092, Sigma-Aldrich) and incubated with membranes overnight. β-Actin (1:10,000 a3854, Sigma-Aldrich) levels were measured as a loading control. Horseradish peroxidase (HRP)-conjugated anti-rabbit IgG (1:10,000, A6154, Sigma- Aldrich) was used as the secondary antibody. Immunoreactivity was detected using chemiluminescent HRP substrate and blots were imaged on a ChemiDoc MP imager. Densitometry using ImageJ (v1.51 NIH)[53] was used to quantify signals in relevant bands. Band intensities for relevant proteins were normalized to the signal from β-actin, then to the average signal from WT mice, and are displayed as relative changes from WT. The samples were not blinded to the performer of the analysis.

Flow cytometry analysis

The mice were euthanized by CO_2 narcosis. Mouse spleens were removed and placed into a 1.5-mL microfuge tube with cold sterile PBS on ice. Total BM was collected as described above and placed on ice. To generate a single-cell suspension from the spleen, the organs were placed in a sterile 100-μm cell strainer mesh in a 50-mL conical tube. Using the plunger of a 1-mL syringe, the organs were mashed until very fine parts remained. The strainers were washed with a flushing buffer (3% FBS, 0.2 mM EDTA in PBS). Both BM and spleen cells were then centrifuged (500 g, 10 min, 4°C) and supernatants were removed. After washing with PBS containing 0.5% BSA and 0.05% sodium azide, the cells were stained with fluorescence-conjugated antibodies fluorescein isothiocyanate (FITC) anti-mouse

CD45 (cn: 103108), APC anti-mouse CD11b (cn: 101201), PE anti-mouse CD3 (cn: 100206), and Brilliant Violet 421 anti-mouse CD19 (cn: 115549, all from BioLegend) for 30 min at 4°C, washed, and analyzed using an Aurora flow cytometer (BD Bioscience, San Jose, CA). CD11b+ and CD19+ cells were selected from the CD45+ population. All samples were blinded to the performer of the analysis.

Mass spectrometry

Mouse tissues were processed prior to lipid extraction by mechanical homogenization in an Ultra-Turrax (Miccra D-9, MICCRA GmbH, Heitersheim, Germany) in MilliQ water. Final homogenate concentrations were in the range of 5%–25% according to the wet weight (wwt) of the tissue samples in water. Protein content in homogenates was measured by the method of Hartree.[54] Extraction of Cer, CMH, CDH, SM, DHC, and SPH and GM1, GM2, and GM3 gangliosides was performed from 10 μL of homogenate by following a previously published method[55] using chloroform:methanol (2:1, v/v). The S1P and C1P were extracted from 50 μL of homogenate by following a previously published method.[55]

Lipids were analyzed on AB/MDS SCIEX API4000 triple quadrupole tandem mass spectrometer with electrospray ionization (ESI; Applied Biosystems, Waltham, MA) coupled to an Agilent UPLC Infinity 1290 system equipped with an autosampler (Agilent Technologies, Santa Clara, CA). All samples were measured in technical replicates and average values were calculated for each sample.

Lipids were analyzed by two approaches. Cer, monohexosylceramides (MHCs), DHC, sphingomyelin (SM), and monosialogangliosides GM1, GM2, and GM3 were analyzed by flow injection electrospray ionization tandem mass spectrometry (FIA-ESI-MS/MS) using methanol as a mobile phase.[56,57] Liquid chromatography combined with electrospray ionization tandem mass spectrometry LC-ESI-MS/MS was used for the analysis of DHC, SPH, and C1P based on slightly modified previously published methods.[55] The C1P transition pairs were added to the existing method for analysis of S1P and optimized ion optic parameters for C1P were set as follows: decluttering potential 69 V, entrance potential 11 V, collision energy 43, and collision cell exit potential 11 V. The measured ions for C1P set as m/z were 618.7, 646.7, 730.7, 620.7, 648.7, and 592.7 for IST with common fragment for all ions 264.4 m/z. Dwell time for all ion signal collection was 1,000 ms per ion. The DHC and SPH were analyzed using modified method for liquid chromatography-mass spectrometry (LC-MS) analysis of Cer and SPH by replacing the transition pairs for Cer by values elevated by 2 m/z to match the additional two hydrogen atoms in dihydroceramide and using the product ion with 284.2 m/z[55] The column with denser Silica-C silica gel (Cogent Silica-C 2.0 column, 2.2 μm 120 A 75 × 2.1 mm, no. 40200-75-P-2 from Microsolv, Leland, NC, USA) and higher flow rate of 200 μL/min was used.

FIA-ESI-MS/MS and LC-ESI-MS/MS used for analysis of other lipids was based on previously published methods.[56,57] Results were analyzed using Analyst 1.6.3 software (Applied Biosystems, Waltham, MA, USA). The concentration of these lipids was calculated according to previously published methods by single-point calibration with a known lipid concentration in external calibration standard corrected by the signal ratio toward internal standard (IST).[56] SPH concentration was calculated directly from ratio of measured molecular species toward the signal of IST.[55] Final concentrations of each lipid class or molecular species were expressed as picomoles of analyzed lipid per milligram of protein. Molecular species (isoform) profiles were calculated using measured signals of individual molecules. Final concentrations there were also expressed as picomoles of lipids per milligram of protein. All samples were blinded to the performer of the analysis.

Creatinine and BUN assay

Urine samples of treated and untreated P361R-FD, P361R-SMA, and WT mice were collected using hydrophobic LabSand (Coastine Global, West Chester, PA, USA) placed inside the cages. All samples were collected between 8 am and 3 pm, briefly centrifuged (100 × g), aliquoted, and stored at −80°C until further use. Creatinine assays were performed using Creatinine Assay Kit (LS-K207, LS Bio, Seattle, WA, USA) in accordance with the manufacturer's instructions. A urea nitrogen assay was performed on the same samples using a BUN kit (K024-H1, Arbor Assay, Ann Arbor, MI, USA) in accordance with the manufacturer's instructions. All samples were blinded to the performer of the analysis.

Statistical analyses

Most experiments were conducted with at least three replicates. Statistical tests used for each experiment to report significance are indicated in the figure legends. All statistics were analyzed using GraphPad Prism 5.0 (GraphPad Software, La Jolla, CA). Significant differences are expressed as $^{*}p < 0.05$, $^{**}p < 0.01$, $^{***}p < 0.001$, and $^{****}p < 0.0001$.

DATA AND CODE AVAILABILITY

All data are included in the paper or the supplemental information. Additional data are available from the corresponding authors on reasonable request.

SUPPLEMENTAL INFORMATION

Supplemental information can be found online at https://doi.org/10.1016/j.ymthe.2024.08.004.

ACKNOWLEDGMENTS

This work was supported by the Midwest Athletes Against Childhood Cancer (MACC) Fund Professorship to J.A.M. This work was also supported by the Department of Pediatrics at MCW. In addition, some support was provided by the Ministry of Health of the Czech Republic, grant no. NU21-08-00324, by the project National Institute for Research of Metabolic and Cardiovascular Diseases (Programme EXCELES, ID project no. LX22NPO5104), funded by the European Union - Next Generation EU and the Institutional Programs of Charles University in Prague UNCE/MED/007 and Cooperatio/DIAG/1FM.

We thank Christine Duris and the Children's Research Institute Histology Core for technical support, Dr. Suresh Kumar and the Children's Research Institute Imaging Core for help with microscopy, along with Jennifer Sterrett and the MCW Neuroscience Research Center for help with the behavioral studies.

AUTHOR CONTRIBUTIONS

Conceptualization, J.R.; methodology, J.R., T.S., L.K., P.R., and T.D., Investigation, J.R.; visualization, J.R. and W.M.M.; supervision, J.A.M.; writing – original draft, J.R.; writing – review & editing; J.R., T.S., L.K., T.D., W.M.M., and J.A.M.

DECLARATION OF INTERESTS

The authors declare no competing interests.

DECLARATION OF GENERATIVE AI AND AI-ASSISTED TECHNOLOGIES IN THE WRITING PROCESS

During the preparation of this work the authors did not used any generative AI or AI-assisted technologies in the writing process.

REFERENCES

1. Yu, F.P.S., Amintas, S., Levade, T., and Medin, J.A. (2018). Acid ceramidase deficiency: Farber disease and SMA-PME. Orphanet J. Rare Dis. *13*, 121. https://doi.org/10.1186/s13023-018-0845-z.

2. Antonarakis, S.E., Valle, D., Moser, H.W., Moser, A., Qualman, S.J., and Zinkham, W.H. (1984). Phenotypic variability in siblings with Farber disease. J. Pediatr. *104*, 406–409. https://doi.org/10.1016/s0022-3476(84)81106-3.

3. Ehlert, K., Frosch, M., Fehse, N., Zander, A., Roth, J., and Vormoor, J. (2007). Farber disease: clinical presentation, pathogenesis and a new approach to treatment. Pediatr. Rheumatol. Online J. *5*, 15. https://doi.org/10.1186/1546-0096-5-15.

4. Najafi, A., Tasharrofi, B., Zandsalimi, F., Rasulinezhad, M., Ghahvechi Akbari, M., Zamani, G., Ashrafi, M.R., and Heidari, M. (2023). Spinal Muscular Atrophy with Progressive Myoclonic Epilepsy (SMA-PME): three new cases and review of the mutational spectrum. Ital. J. Pediatr. *49*, 64. https://doi.org/10.1186/s13052-023-01474-z.

5. Kleynerman, A., Rybova, J., Faber, M.L., McKillop, W.M., Levade, T., and Medin, J.A. (2023). Acid Ceramidase Deficiency: Bridging Gaps between Clinical Presentation, Mouse Models, and Future Therapeutic Interventions. Biomolecules *13*, 274. https://doi.org/10.3390/biom13020274.

6. Dyment, D.A., Sell, E., Vanstone, M.R., Smith, A.C., Garandeau, D., Garcia, V., Carpentier, S., Le Trionnaire, E., Sabourdy, F., Beaulieu, C.L., et al. (2014). Evidence for clinical, genetic and biochemical variability in spinal muscular atrophy with progressive myoclonic epilepsy. Clin. Genet. *86*, 558–563. https://doi.org/10.1111/cge.12307.

7. Lee, M.M., McDowell, G.S.V., De Vivo, D.C., Friedman, D., Berkovic, S.F., Spanou, M., Dinopoulos, A., Grand, K., Sanchez-Lara, P.A., Allen-Sharpley, M., et al. (2022). The clinical spectrum of SMA-PME and *in vitro* normalization of its cellular ceramide profile. Ann. Clin. Transl. Neurol. *9*, 1941–1952. https://doi.org/10.1002/acn3.51687.

8. Zhou, H., Wu, Z., Wang, Y., Wu, Q., Hu, M., Ma, S., Zhou, M., Sun, Y., Yu, B., Ye, J., et al. (2022). Rare Diseases in Glycosphingolipid Metabolism. Adv. Exp. Med. Biol. *1372*, 189–213. https://doi.org/10.1007/978-981-19-0394-6_13.

9. Rossini, L., Durante, C., Marzollo, A., and Biffi, A. (2022). New Indications for Hematopoietic Stem Cell Gene Therapy in Lysosomal Storage Disorders. Front. Oncol. *12*, 885639. https://doi.org/10.3389/fonc.2022.885639.

10. Thomas, E.D., Lochte, H.L., Jr., Lu, W.C., and Ferrebee, J.W. (1957). Intravenous infusion of bone marrow in patients receiving radiation and chemotherapy. N. Engl. J. Med. *257*, 491–496. https://doi.org/10.1056/NEJM195709122571102.

11. Lund, T.C. (2013). Hematopoietic Stem Cell Transplant for Lysosomal Storage Diseases. Pediatr. Endocr. Rev. P *11*, 91–98.

12. Bigger, B.W., and Wynn, R.F. (2014). Novel Approaches and Mechanisms in Hematopoietic Stem Cell Gene Therapy. Discov. Med. *17*, 207–215.

13. Tan, E.Y., Boelens, J.J., Jones, S.A., and Wynn, R.F. (2019). Hematopoietic Stem Cell Transplantation in Inborn Errors of Metabolism. Front. Pediatr. *7*, 433. https://doi.org/10.3389/fped.2019.00433.

14. Tomatsu, S., Taylor, M., and Khan, S. (2019). Hematopoietic stem cell transplantation for mucopolysaccharidoses past, present, and future. Mol. Genet. Metab. *126*, S145. https://doi.org/10.1016/j.ymgme.2018.12.375.

15. Ehlert, K., Roth, J., Frosch, M., Fehse, N., Zander, N., and Vormoor, J. (2006). Farber's disease without central nervous system involvement: bone-marrow transplantation provides a promising new approach. Ann. Rheum. Dis. *65*, 1665–1666. https://doi.org/10.1136/ard.2005.048322.

16. Broomfield, A.A., Chakrapani, A., and Wraith, J.E. (2010). The effects of early and late bone marrow transplantation in siblings with alpha-mannosidosis. Is early haematopoietic cell transplantation the preferred treatment option? J. Inherit. Metab. Dis. *33*, S123–S127. https://doi.org/10.1007/s10545-009-9035-4.

17. Escolar, M.L., Poe, M.D., Provenzale, J.M., Richards, K.C., Allison, J., Wood, S., Wenger, D.A., Pietryga, D., Wall, D., Champagne, M., et al. (2005). Transplantation of umbilical-cord blood in babies with infantile Krabbe's disease. N. Engl. J. Med. *352*, 2069–2081. https://doi.org/10.1056/NEJMoa042604.

18. Goudie, C., Alayoubi, A.M., Tibout, P., Duval, M., Maranda, B., Mitchell, D., and Mitchell, J.J. (2019). Hematopoietic stem cell transplant does not prevent neurological deterioration in infants with Farber disease: Case report and literature review. JIMD Rep. *46*, 46–51. https://doi.org/10.1002/jmd2.12008.

19. Sikora, J., Dworski, S., Jones, E.E., Kamani, M.A., Micsenyi, M.C., Sawada, T., Le Faouder, P., Bertrand-Michel, J., Dupuy, A., Dunn, C.K., et al. (2017). Acid Ceramidase Deficiency in Mice Results in a Broad Range of Central Nervous System Abnormalities. Am. J. Pathol. *187*, 864–883. https://doi.org/10.1016/j.ajpath.2016.12.005.

20. Yu, F.P.S., Sajdak, B.S., Sikora, J., Salmon, A.E., Nagree, M.S., Gurka, J., Kassem, I.S., Lipinski, D.M., Carroll, J., and Medin, J.A. (2019). Acid Ceramidase Deficiency in Mice Leads to Severe Ocular Pathology and Visual Impairment. Am. J. Pathol. *189*, 320–338. https://doi.org/10.1016/j.ajpath.2018.10.018.

21. Yu, F.P.S., Islam, D., Sikora, J., Dworski, S., Gurka, J., López-Vásquez, L., Liu, M., Kuebler, W.M., Levade, T., Zhang, H., and Medin, J.A. (2018). Chronic lung injury and impaired pulmonary function in a mouse model of acid ceramidase deficiency. Am. J. Physiol. Lung Cell. Mol. Physiol. *314*, L406–L420. https://doi.org/10.1152/ajplung.00223.2017.

22. Dworski, S., Berger, A., Furlonger, C., Moreau, J.M., Yoshimitsu, M., Trentadue, J., Au, B.C.Y., Paige, C.J., and Medin, J.A. (2015). Markedly perturbed hematopoiesis in acid ceramidase deficient mice. Haematologica *100*, e162–e165. https://doi.org/10.3324/haematol.2014.108530.

23. Rybova, J., Kuchar, L., Sikora, J., McKillop, W.M., and Medin, J.A. (2022). Skin inflammation and impaired adipogenesis in a mouse model of acid ceramidase deficiency. J. Inherit. Metab. Dis. *45*, 1175–1190. https://doi.org/10.1002/jimd.12552.

24. Yu, F.P.S., Molino, S., Sikora, J., Rasmussen, S., Rybova, J., Tate, E., Geurts, A.M., Turner, P.V., McKillop, W.M., and Medin, J.A. (2019). Hepatic pathology and altered gene transcription in a murine model of acid ceramidase deficiency. Lab. Invest. *99*, 1572–1592. https://doi.org/10.1038/s41374-019-0271-4.

25. Alayoubi, A.M., Wang, J.C.M., Au, B.C.Y., Carpentier, S., Garcia, V., Dworski, S., El-Ghamrasni, S., Kirouac, K.N., Exertier, M.J., Xiong, Z.J., et al. (2013). Systemic ceramide accumulation leads to severe and varied pathological consequences. EMBO Mol. Med. *5*, 827–842. https://doi.org/10.1002/emmm.201202301.

26. Nagree, M.S., Rybova, J., Kleynerman, A., Ahrenhoerster, C.J., Saville, J.T., Xu, T., Bachochin, M., McKillop, W.M., Lawlor, M.W., Pshezhetsky, A.V., et al. (2023). Spinal muscular atrophy-like phenotype in a mouse model of acid ceramidase deficiency. Commun. Biol. *6*, 560. https://doi.org/10.1038/s42003-023-04932-w.

27. Dworski, S., Lu, P., Khan, A., Maranda, B., Mitchell, J.J., Parini, R., Di Rocco, M., Hugle, B., Yoshimitsu, M., Magnusson, B., et al. (2017). Acid Ceramidase Deficiency is characterized by a unique plasma cytokine and ceramide profile that

is altered by therapy. BBA - Mol. Basis Dis. *1863*, 386–394. https://doi.org/10.1016/j.bbadis.2016.11.031.

28. Beckmann, N., Kadow, S., Schumacher, F., Göthert, J.R., Kesper, S., Draeger, A., Schulz-Schaeffer, W.J., Wang, J., Becker, J.U., Kramer, M., et al. (2018). Pathological manifestations of Farber disease in a new mouse model. Biol. Chem. *399*, 1183–1202. https://doi.org/10.1515/hsz-2018-0170.
29. Cappellari, A.M., Torcoletti, M., Triulzi, F., and Corona, F. (2016). Nervous system involvement in Farber disease. J. Inherit. Metab. Dis. *39*, 149–150. https://doi.org/10.1007/s10545-015-9890-0.
30. Zielonka, M., Garbade, S.F., Kölker, S., Hoffmann, G.F., and Ries, M. (2018). A cross-sectional quantitative analysis of the natural history of Farber disease: an ultra-orphan condition with rheumatologic and neurological cardinal disease features. Genet. Med. *20*, 524–530. https://doi.org/10.1038/gim.2017.133.
31. Wu, N.L., Hingorani, S., Cushing-Haugen, K.L., Lee, S.J., and Chow, E.J. (2021). Late Kidney Morbidity and Mortality in Hematopoietic Cell Transplant Survivors. Transpl. Cell. Ther. *27*, 434.e1–434.e6. https://doi.org/10.1016/j.jtct.2021.02.013.
32. Hingorani, S., Guthrie, K.A., Schoch, G., Weiss, N.S., and McDonald, G.B. (2007). Chronic kidney disease in long-term survivors of hematopoietic cell transplant. Bone Marrow Transpl. *39*, 223–229. https://doi.org/10.1038/sj.bmt.1705573.
33. Wynn, R.F., Wraith, J.E., Mercer, J., O'Meara, A., Tylee, K., Thornley, M., Church, H.J., and Bigger, B.W. (2009). Improved metabolic correction in patients with lysosomal storage disease treated with hematopoietic stem cell transplant compared with enzyme replacement therapy. J. Pediatr. *154*, 609–611. https://doi.org/10.1016/j.jpeds.2008.11.005.
34. Torcoletti, M., Petaccia, A., Pinto, R.M., Hladnik, U., Locatelli, F., Agostoni, C., and Corona, F. (2014). Farber disease in infancy resembling juvenile idiopathic arthritis: identification of two new mutations and a good early response to allogeneic haematopoietic stem cell transplantation. Rheumatology (Oxford) *53*, 1533–1534. https://doi.org/10.1093/rheumatology/keu010.
35. Zappatini-Tommasi, L., Dumontel, C., Guibaud, P., and Girod, C. (1992). Farber disease: an ultrastructural study. Report of a case and review of the literature. Virchows Arch. A. Pathol. Anat. Histopathol. *420*, 281–290. https://doi.org/10.1007/BF01600282.
36. Singh, S., Anshita, D., and Ravichandiran, V. (2021). MCP-1: Function, regulation, and involvement in disease. Int. Immunopharmacol. *101*, 107598. https://doi.org/10.1016/j.intimp.2021.107598.
37. Solomon, M., and Muro, S. (2017). Lysosomal enzyme replacement therapies: Historical development, clinical outcomes, and future perspectives. Adv. Drug Deliv. Rev. *118*, 109–134. https://doi.org/10.1016/j.addr.2017.05.004.
38. Fratantoni, J.C., Neufeld, E.F., Uhlendorf, B.W., and Jacobson, C.B. (1969). Intrauterine diagnosis of the hurler and hunter syndromes. N. Engl. J. Med. *280*, 686–688. https://doi.org/10.1056/NEJM196903272801303.
39. Edelmann, M.J., and Maegawa, G.H.B. (2020). CNS-Targeting Therapies for Lysosomal Storage Diseases: Current Advances and Challenges. Front. Mol. Biosci. *7*, 559804. https://doi.org/10.3389/fmolb.2020.559804.
40. Schuchman, E.H., He, X., Dworski, S., Zhu, C., DeAngelis, V., Solyom, A., Levade, T., Medin, J.A., and Simonaro, C.M. (2017). Proof-of-concept studies underlying enzyme replacement therapy for acid ceramidase deficiency. Mol. Genet. Metab. *120*, S120. https://doi.org/10.1016/j.ymgme.2016.11.310.
41. Mitchell, J., Solyom, A., Makay, B., Arslan, N., Batu, E.D., Ozen, S., Hügle, B., Schuchman, E., and Magnusson, B. (2016). Farber disease: Implications of anti-inflammatory treatment. Mol. Genet. Metab. *117*, S81–S82. https://doi.org/10.1016/j.ymgme.2015.12.364.
42. Vormoor, J., Ehlert, K., Groll, A.H., Koch, H.G., Frosch, M., and Roth, J. (2004). Successful hematopoietic stem cell transplantation in Farber disease. J. Pediatr. *144*, 132–134. https://doi.org/10.1016/j.jpeds.2003.09.051.
43. Lee, M.M., McDowell, G.S.V., De Vivo, D.C., Friedman, D., Berkovic, S.F., Spanou, M., Dinopoulos, A., Grand, K., Sanchez-Lara, P.A., Allen-Sharpley, M., et al. (2022). The clinical spectrum of SMA-PME and *in vitro* normalization of its cellular ceramide profile. Ann. Clin. Transl. Neurol. *9*, 1941–1952. https://doi.org/10.1002/acn3.51687.
44. Mohseni, R., Hamidieh, A.A., Shoae-Hassani, A., Ghahvechi-Akbari, M., Majma, A., Mohammadi, M., Nikougoftar, M., Shervin-Badv, R., Ai, J., Montazerlotfelahi, H., and Ashrafi, M.R. (2022). An open-label phase 1 clinical trial of the allogeneic side population adipose-derived mesenchymal stem cells in SMA type 1 patients. Neurol. Sci. *43*, 399–410. https://doi.org/10.1007/s10072-021-05291-2.
45. Cabanes, C., Bonilla, S., Tabares, L., and Martínez, S. (2007). Neuroprotective effect of adult hematopoietic stem cells in a mouse model of motoneuron degeneration. Neurobiol. Dis. *26*, 408–418. https://doi.org/10.1016/j.nbd.2007.01.008.
46. Li, G., Kidd, J., Kaspar, C., Dempsey, S., Bhat, O.M., Camus, S., Ritter, J.K., Gehr, T.W.B., Gulbins, E., and Li, P.L. (2020). Podocytopathy and Nephrotic Syndrome in Mice with Podocyte-Specific Deletion of the Asah1 Gene: Role of Ceramide Accumulation in Glomeruli. Am. J. Pathol. *190*, 1211–1223. https://doi.org/10.1016/j.ajpath.2020.02.008.
47. Renaghan, A.D., Jaimes, E.A., Malyszko, J., Perazella, M.A., Sprangers, B., and Rosner, M.H. (2020). Acute Kidney Injury and CKD Associated with Hematopoietic Stem Cell Transplantation. Clin. J. Am. Soc. Nephrol. *15*, 289–297. https://doi.org/10.2215/CJN.08580719.
48. Rodrigues, N., Fragao-Marques, M., Costa, C., Branco, C., Marques, F., Vasconcelos, P., Martins, C., Leite-Moreira, A., and Lopes, J.A. (2023). Predictive Risk Score for Acute Kidney Injury in Hematopoietic Stem Cell Transplant. Cancers (Basel) *15*, 3720. https://doi.org/10.3390/cancers15143720.
49. Kong, S.G., Jeong, S., Lee, S., Jeong, J.Y., Kim, D.J., and Lee, H.S. (2021). Early transplantation-related mortality after allogeneic hematopoietic cell transplantation in patients with acute leukemia. BMC Cancer *21*, 177. https://doi.org/10.1186/s12885-021-07897-3.
50. Savas, B., Astarita, G., Aureli, M., Sahali, D., and Ollero, M. (2020). Gangliosides in Podocyte Biology and Disease. Int. J. Mol. Sci. *21*, 9645. https://doi.org/10.3390/ijms21249645.
51. Masson, E., Wiernsperger, N., Lagarde, M., and El Bawab, S. (2005). Glucosamine induces cell-cycle arrest and hypertrophy of mesangial cells: implication of gangliosides. Biochem. J. *388*, 537–544. https://doi.org/10.1042/BJ20041506.
52. Liu, X., and Quan, N. (2015). Immune Cell Isolation from Mouse Femur Bone Marrow. Bio. Protoc. *5*, e1631. https://doi.org/10.21769/bioprotoc.1631.
53. Schneider, C.A., Rasband, W.S., and Eliceiri, K.W. (2012). NIH Image to ImageJ: 25 years of image analysis. Nat Methods *9*, 671–675. https://doi.org/10.1038/nmeth.2089.
54. Hartree, E.F. (1972). Determination of protein: a modification of the Lowry method that gives a linear photometric response. Anal. Biochem. *48*, 422–427. https://doi.org/10.1016/0003-2697(72)90094-2.
55. Bugajev, V., Paulenda, T., Utekal, P., Mrkacek, M., Halova, I., Kuchar, L., Kuda, O., Vavrova, P., Schuster, B., Fuentes-Liso, S., et al. (2021). Crosstalk between ORMDL3, serine palmitoyltransferase, and 5-lipoxygenase in the sphingolipid and eicosanoid metabolic pathways. J. Lipid Res. *62*, 100121. https://doi.org/10.1016/j.jlr.2021.100121.
56. Kuchar, L., Ledvinová, J., Hrebícek, M., Mysková, H., Dvoráková, L., Berná, L., Chrastina, P., Asfaw, B., Elleder, M., Petermöller, M., et al. (2009). Prosaposin deficiency and saposin B deficiency (activator-deficient metachromatic leukodystrophy): report on two patients detected by analysis of urinary sphingolipids and carrying novel PSAP gene mutations. Am. J. Med. Genet. A. *149A*, 613–621. https://doi.org/10.1002/ajmg.a.32712.
57. Hulkova, H., Ledvinova, J., Kuchar, L., Smid, F., Honzikova, J., and Elleder, M. (2012). Glycosphingolipid profile of the apical pole of human placental capillaries: the relevancy of the observed data to Fabry disease. Glycobiology *22*, 725–732. https://doi.org/10.1093/glycob/cws050.

YMTHE, Volume *32*

Supplemental Information

Hematopoietic stem cell transplantation leads to biochemical and functional correction in two mouse models of acid ceramidase deficiency

Jitka Rybova, Teresa Sundararajan, Ladislav Kuchar, Theresa A. Dlugi, Petr Ruzicka, William M. McKillop, and Jeffrey A. Medin

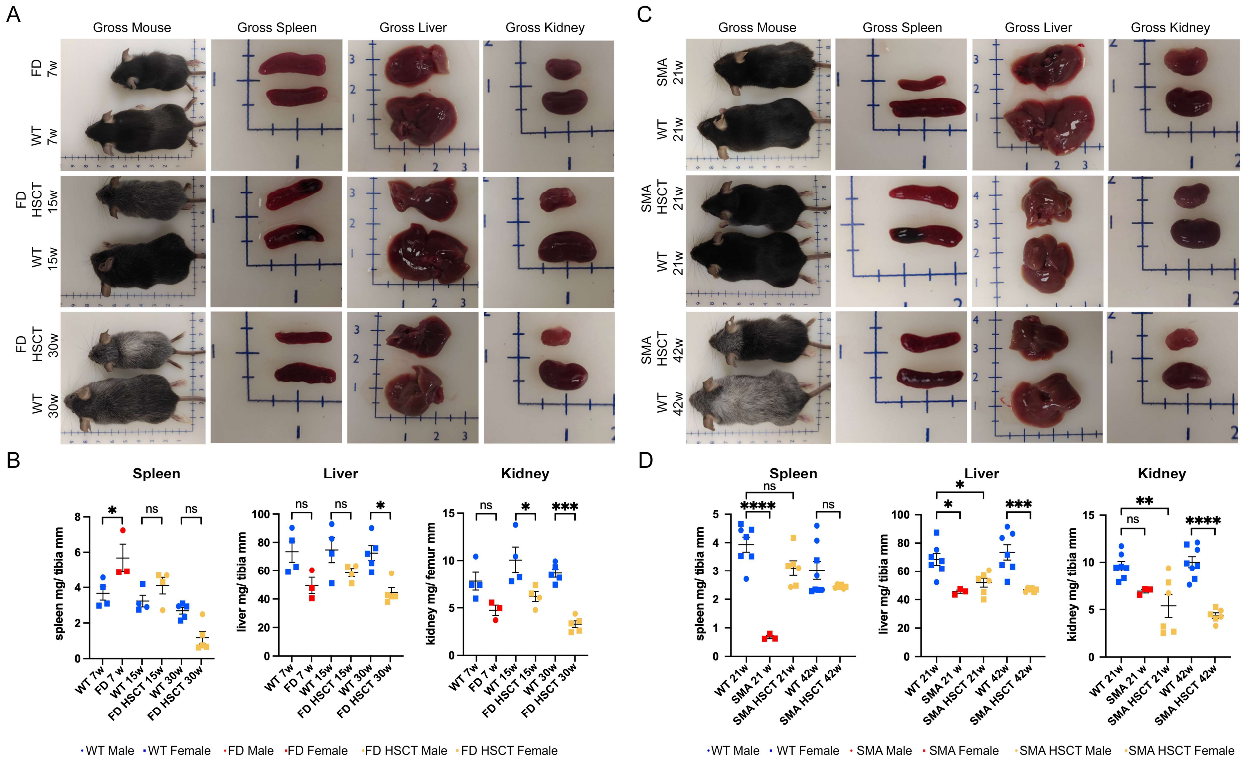

Figure S1: Body and organ weight variation following HSCT in P361R-FD mice and P361R-SMA mice

(A) Gross body, spleen, liver, and kidney images of females from untreated P361R-FD mice (7-week-old), HSC-transplanted P361R-FD mice (15- and 30-week-old), and WT mice (7-, 15- and 30-week-old).

(B) Organ weights of untreated P361R-FD mice (7-weeks-old), HSC-transplanted P361R-FD mice (15- and 30-week-old), and WT mice (7-, 15- and 30-week-old) (n = 3-5 mice per group, biological replicates). Standard error depicted. Gender combined for statistical analysis. Data analyzed using one-way ANOVA with Tukey's multiple comparison test. Significant results: *p < 0.05; ***p < 0.001, ns = not significant.

(C) Gross body, spleen, liver, and kidney images from female untreated P361R-SMA mice (21-week-old), HSC-transplanted P361R-SMA mice (21- and 42-week-old), and WT mice (21- and 42-week-old).

(D) Organ weights of untreated P361R-SMA mice (21-week-old), HSC-transplanted P361R-SMA mice (21- and 42-week-old), and WT mice (21- and 42-week-old) (n = 3-5 mice per group, biological replicates). Standard error depicted. Gender combined for statistical analysis. Data analyzed using one-way ANOVA with Tukey's multiple comparison test. Significant results: $^{*}p < 0.05$; $^{**}p < 0.01$; $^{***}p < 0.001$; $^{****}p < 0.0001$, ns = not significant.

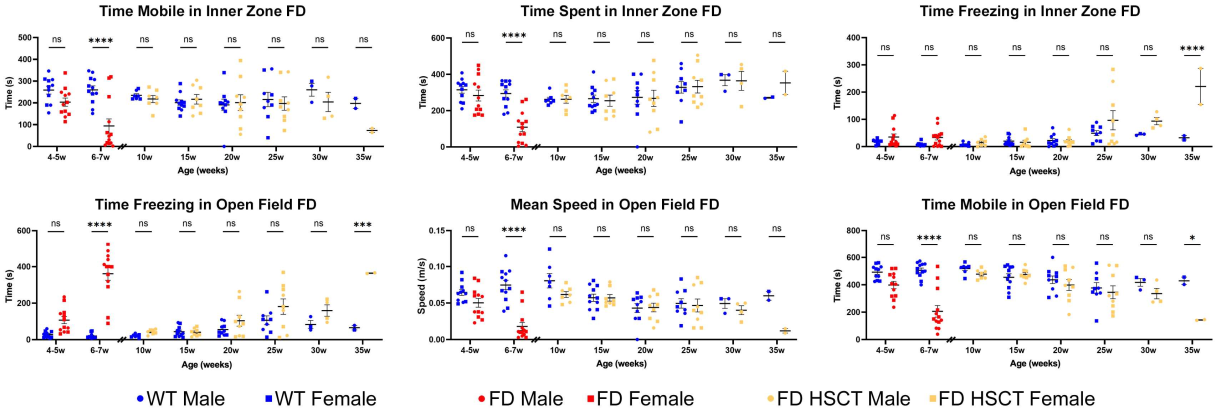

Figure S2: Open field behavioral characteristics of P361R-FD mice following HSCT

Open field characteristics including: time mobile in the inner zone, total time spent in the inner zone, time spent freezing in the inner zone, time spent freezing in the total open field, mean speed of travel in the open field, and time spent mobile in the entire open field was evaluated in untreated P361R-FD mice and WT control mice (at 4-5 and 6-7 weeks of age) in comparison to HSC-transplanted P361R-FD mice and age-matched WT littermates (n = 11-13 for each group, biological replicates). Standard error depicted. Gender combined for statistical analysis. Data analyzed using Sidak's multiple comparison test between WT and FD mice at 4-5 and 6-7 weeks of age, and between WT and HSCT treated FD mice between 10 and 35 weeks of age. Significant results: *$p < 0.05$; ***$p < 0.001$; ****$p < 0.0001$, ns = not significant.

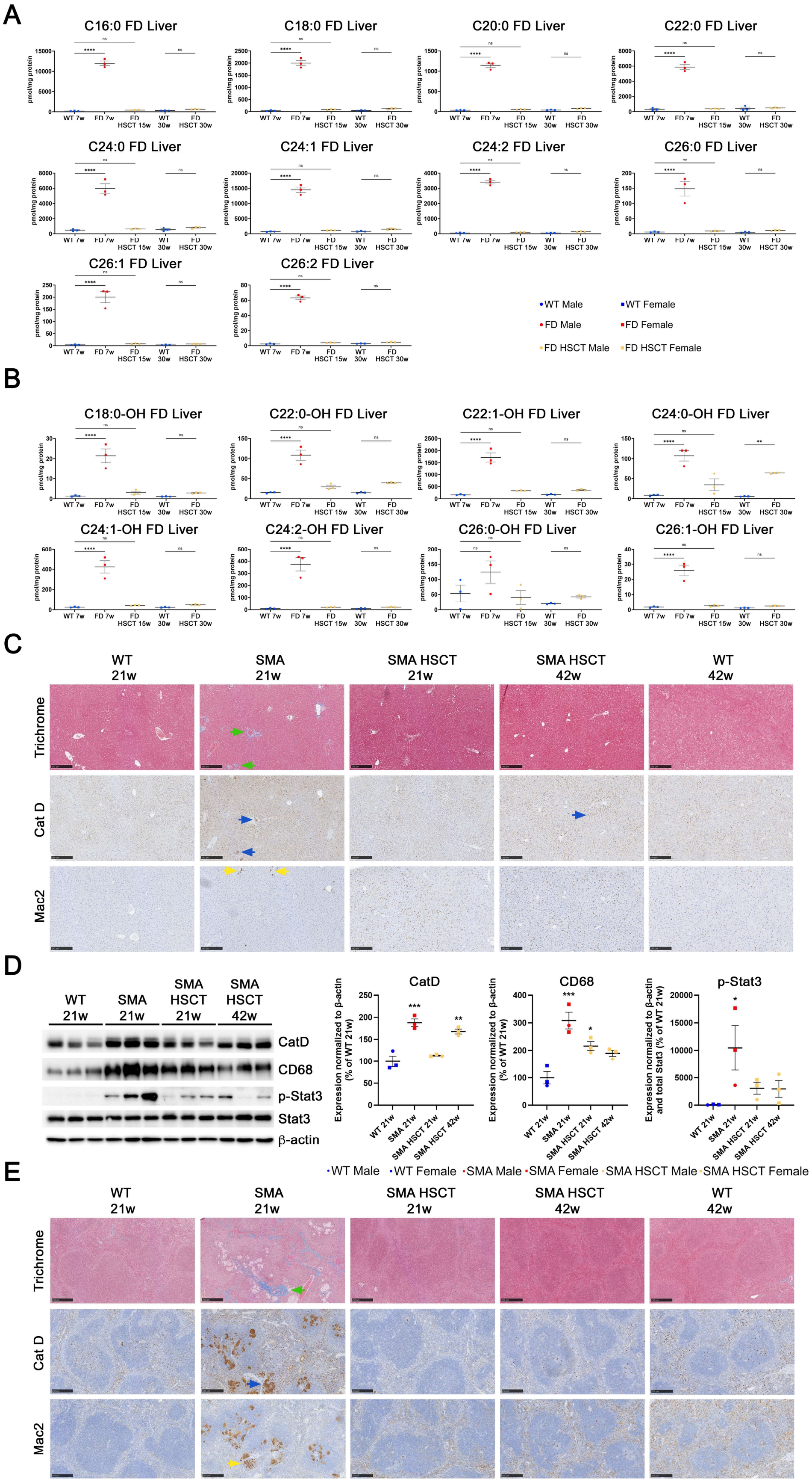
A
C16:0 FD Liver
C18:0 FD Liver
C20:0 FD Liver
C22:0 FD Liver
C24:0 FD Liver
C24:1 FD Liver
C24:2 FD Liver
C26:0 FD Liver
C26:1 FD Liver
C26:2 FD Liver
pmol/mg protein
WT 7w
FD 7w
FD HSCT 15w
WT 30w
FD HSCT 30w
WT Male
WT Female
FD Male
FD Female
FD HSCT Male
FD HSCT Female
B
C18:0-OH FD Liver
C22:0-OH FD Liver
C22:1-OH FD Liver
C24:0-OH FD Liver
C24:1-OH FD Liver
C24:2-OH FD Liver
C26:0-OH FD Liver
C26:1-OH FD Liver
C
WT 21w
SMA 21w
SMA HSCT 21w
SMA HSCT 42w
WT 42w
Trichrome
Cat D
Mac2
D
WT 21w
SMA 21w
SMA HSCT 21w
SMA HSCT 42w
CatD
CD68
p-Stat3
Stat3
β-actin
Expression normalized to β-actin (% of WT 21w)
Expression normalized to β-actin and total Stat3 (% of WT 21w)
WT Male
WT Female
SMA Male
SMA Female
SMA HSCT Male
SMA HSCT Female
E
WT 21w
SMA 21w
SMA HSCT 21w
SMA HSCT 42w
WT 42w
Trichrome
Cat D
Mac2

Figure S3: Liver and spleen pathology in P361R-SMA mice following HSCT

(A-B) Quantification of selected Cer (A) and Cer-OH (B) species differing in fatty acid chain lengths in liver extracts from untreated P361R-FD mice (7-week-old), HSC-transplanted P361R-FD mice (15- and 30-week-old), and age-matched WT mice. Standard error depicted. Gender combined for statistical analysis. Data analyzed using one-way ANOVA with Tukey's multiple comparison test. Significant results: ****$p < 0.0001$, ns = not significant. ($n = 3$ mice per group, biological replicates).

(C) Liver sections from female untreated P361R-SMA mice (21-week-old), HSC-transplanted P361R-SMA mice (21- and 42-week-old), and age-matched WT mice stained by Masson Trichrome or immunostained for macrophages (Galectin-3, Mac-2) or lysosomes (Cathepsin D, CatD). Scale bars indicate 250 um.

(D) Representative immunoblots of Cathepsin D (CatD), CD68, and total and phosphorylated inflammatory protein STAT3 in liver lysates from untreated P361R-SMA mice (21-week-old), HSC-transplanted P361R-SMA mice (21- and 42-week-old), and WT mice (21-week-old) and their quantification (normalized to β-actin) relative to WT mice ($n = 3$ mice per group, biological replicates). Standard error depicted. Gender combined for statistical analysis. Data analyzed using one-way ANOVA with multiple comparison test. Significant results: *$p < 0.05$; **$p < 0.01$; ***$p < 0.001$.

(E) Spleen sections from female untreated P361R-SMA mice (21-week-old), HSC-transplanted P361R-SMA mice (21- and 42-week-old), and age-matched WT mice stained by Masson Trichrome or immunostained for macrophages (Galectin-3, Mac-2) or lysosomes (Cathepsin D, CatD) (E). Scale bars indicate 250 um.

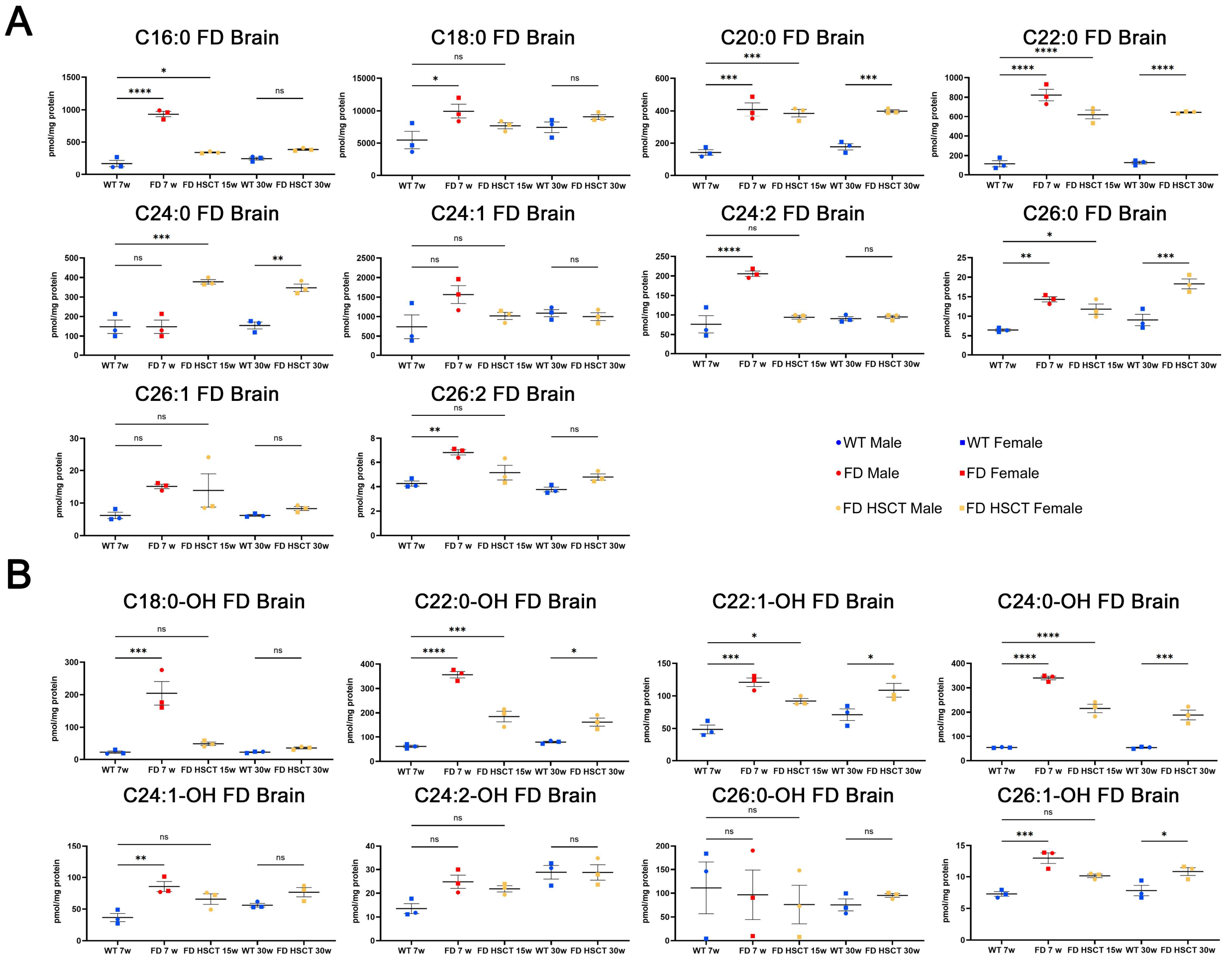

Figure S4: Improvement of brain pathology upon HSCT in both P361R-FD mice

(A, B) Quantification of Cer (A) and Cer-OH (B) species differing in fatty acid chain lengths in brain extracts from untreated P361R-FD mice (7-week-old), HSC-transplanted P361R-FD mice (15- and 30-week-old), and age-matched WT mice (n = 3 mice per group, biological replicates). Standard error depicted. Gender combined for statistical analysis. Data analyzed using one-way ANOVA with Tukey's multiple comparison test. Significant results: *p < 0.05; **p < 0.01; ***p < 0.001; ****P < 0.0001, ns = not significant.

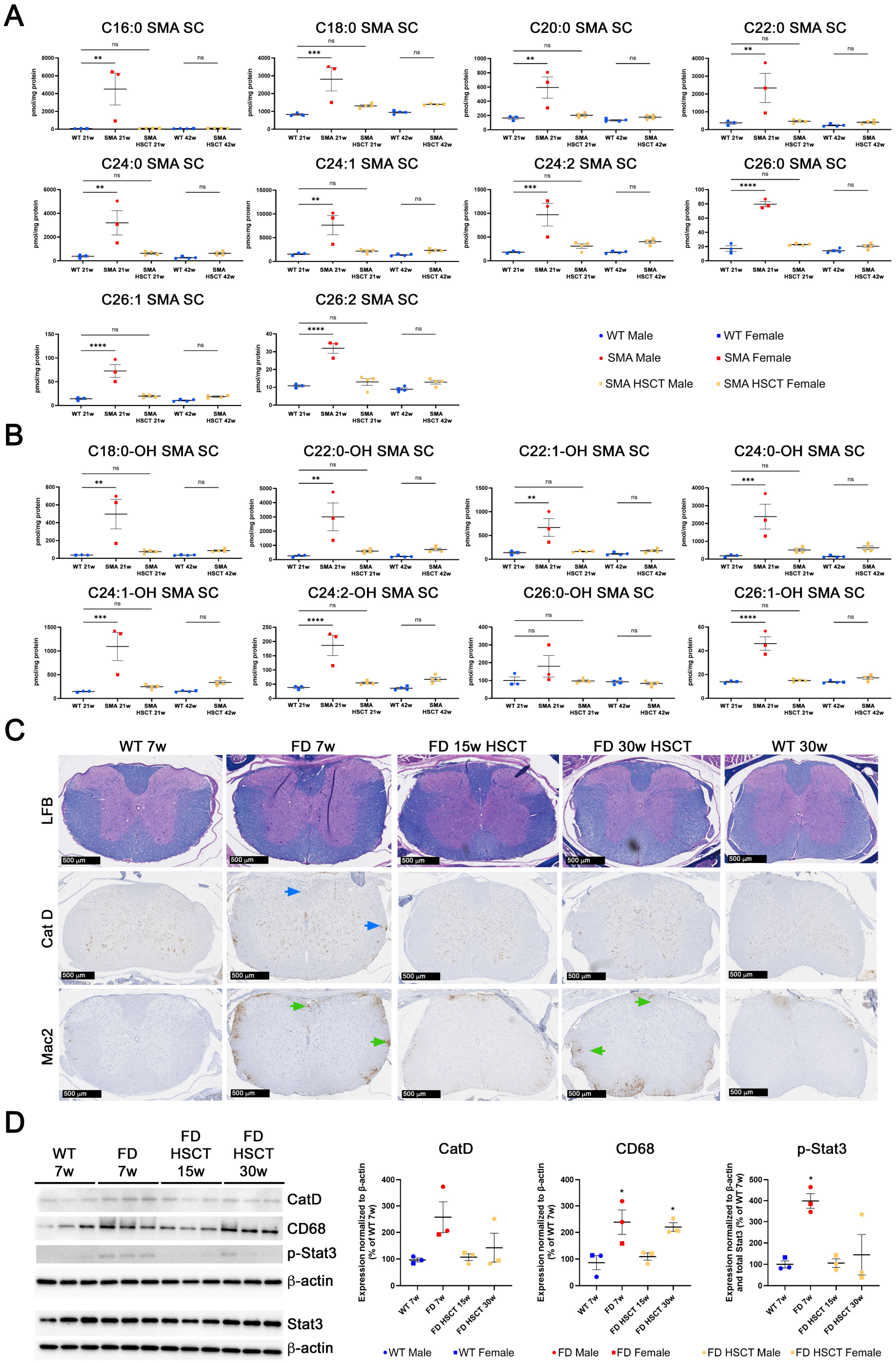
A
C16:0 SMA SC
C18:0 SMA SC
C20:0 SMA SC
C22:0 SMA SC
C24:0 SMA SC
C24:1 SMA SC
C24:2 SMA SC
C26:0 SMA SC
C26:1 SMA SC
C26:2 SMA SC
pmol/mg protein
WT 21w
SMA 21w
SMA HSCT 21w
WT 42w
SMA HSCT 42w
WT Male
WT Female
SMA Male
SMA Female
SMA HSCT Male
SMA HSCT Female
B
C18:0-OH SMA SC
C22:0-OH SMA SC
C22:1-OH SMA SC
C24:0-OH SMA SC
C24:1-OH SMA SC
C24:2-OH SMA SC
C26:0-OH SMA SC
C26:1-OH SMA SC
C
WT 7w
FD 7w
FD 15w HSCT
FD 30w HSCT
WT 30w
LFB
Cat D
Mac2
500 µm
D
WT 7w
FD 7w
FD HSCT 15w
FD HSCT 30w
CatD
CD68
p-Stat3
β-actin
Stat3
β-actin
CatD
CD68
p-Stat3
Expression normalized to β-actin (% of WT 7w)
Expression normalized to β-actin and total Stat3 (% of WT 7w)
WT Male
WT Female
FD Male
FD Female
FD HSCT Male
FD HSCT Female

Figure S5: Normalization of mild spinal cord pathology of P361R-FD and P361R-SMA mice upon HSCT

(A, B) Quantification of Cer (A) and Cer-OH (B) species differing in fatty acid chain lengths in spinal cord (SC) extracts from untreated P361R-SMA mice (21-week-old), HSC-transplanted P361R-SMA mice (21- and 42-week-old), and WT mice (21- and 42-week-old) (n = 3 mice per group, biological replicates). Standard error depicted. Gender combined for statistical analysis. Data analyzed using one-way ANOVA with Tukey's multiple comparison test. Significant results: *$p < 0.05$; **$p < 0.01$; ***$p < 0.001$; ****$P < 0.0001$, ns = not significant.

(C) Spinal cord sections from female untreated P361R-FD mice (7-week-old), HSC-transplanted P361R-FD mice (15- and 30-week-old), and WT mice (7- and 30-week-old) were stained by LFB or immunostained for macrophages (Galectin-3, Mac-2) or lysosomes (Cathepsin D, CatD). Scale bars represent 500 um.

(D) Representative immunoblots of Cathepsin D (CatD), CD68, and total and phosphorylated inflammatory protein STAT3 in liver extracts from untreated P361R-FD mice (7-week-old), HSC-transplanted P361R-FD mice (15- and 30-week-old), and WT mice (7-week-old) and their quantification relative to WT mice (n = 3 mice per group, biological replicates). Standard error depicted. Gender combined for statistical analysis. Data analyzed using one-way ANOVA with multiple comparison test. Significant results: *$p < 0.05$.

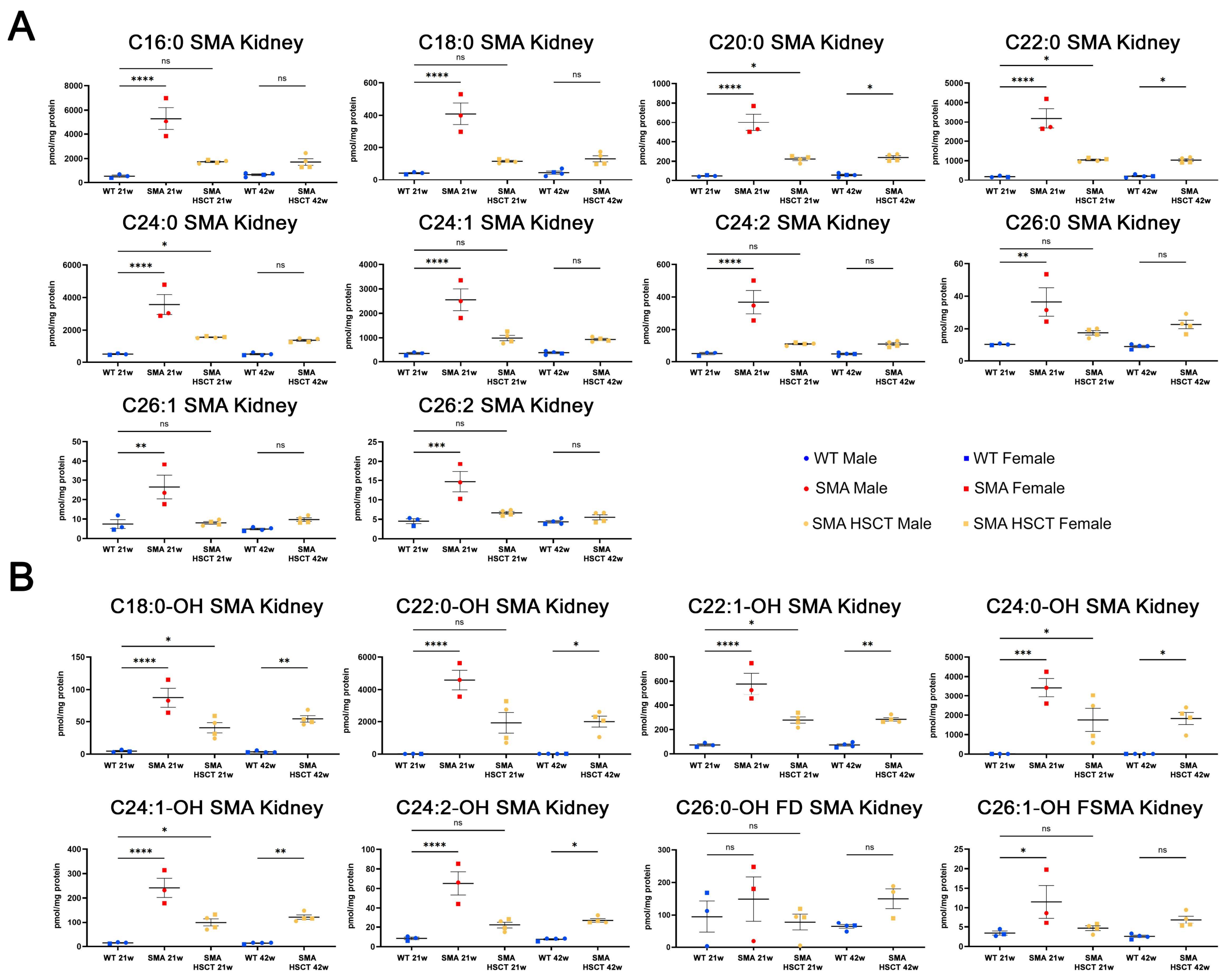

Figure S6: Quantification of ceramide species in kidney extracts from P361R-FD mice and P361R-SMA mice

(A, B) Quantification of Cer (A) and Cer-OH (B) species differing in fatty acid chain lengths in kidney extracts from untreated P361R-SMA mice (21-week-old), HSC-transplanted P361R-SMA mice (21- and 42-week-old), and WT (21- and 42-week-old) mice (n = 3 mice per group, biological replicates). Standard error depicted. Gender combined for statistical analysis. Data analyzed using one-way ANOVA with Tukey's multiple comparison test. Significant results: *p < 0.05; **p < 0.01; ***p < 0.001; ****P < 0.0001, ns = not significant.
